# Recent computational advances in the identification of cryptic binding sites for drug discovery

**DOI:** 10.1093/bioadv/vbaf156

**Published:** 2025-07-01

**Authors:** Dorota Gašparíková, Rupesh Chikhale, Jason Cole, Ehmke Pohl

**Affiliations:** Department of Chemistry, Durham University, Durham DH1 3LE, United Kingdom; Cambridge Crystallographic Data Centre, Cambridge CB2 1EZ, United Kingdom; Cambridge Crystallographic Data Centre, Cambridge CB2 1EZ, United Kingdom; Department of Chemistry, Durham University, Durham DH1 3LE, United Kingdom; Biophysical Sciences Institute, Durham University, Durham DH1 3LE, United Kingdom

## Abstract

**Motivation:**

Cryptic ligand binding sites, defined as binding pockets that exist in the ligand-bound state of a protein but not in its *apo* form, are gaining increasing interest due to the opportunities they provide for drug discovery.

**Results:**

This review article looks at the current state of cryptic binding site research, highlighting advancements in both molecular dynamic (MD) methods and machine learning (ML) methods to predict and utilize these sites.

**Availibilty and Implementation:**

MD methods include the use of Markov State Models, Enhanced Sampling, and other methods such as Cosolvent MD, while ML methods utilize Support Vector Machine, Random Forest, and Neural Networks. Here, we discuss case studies for both methods and their overlaps, providing insight into the future and the limitations faced. Compared to MD methods, ML methods are often reported to be more cost- and time-effective. However, a limited number of datasets are available for training these ML methods. Integrating MD with ML methods promises to expand our ability to predict and validate new cryptic binding sites that can be evaluated for druggability.

## 1 Introduction

**Figure 1.**
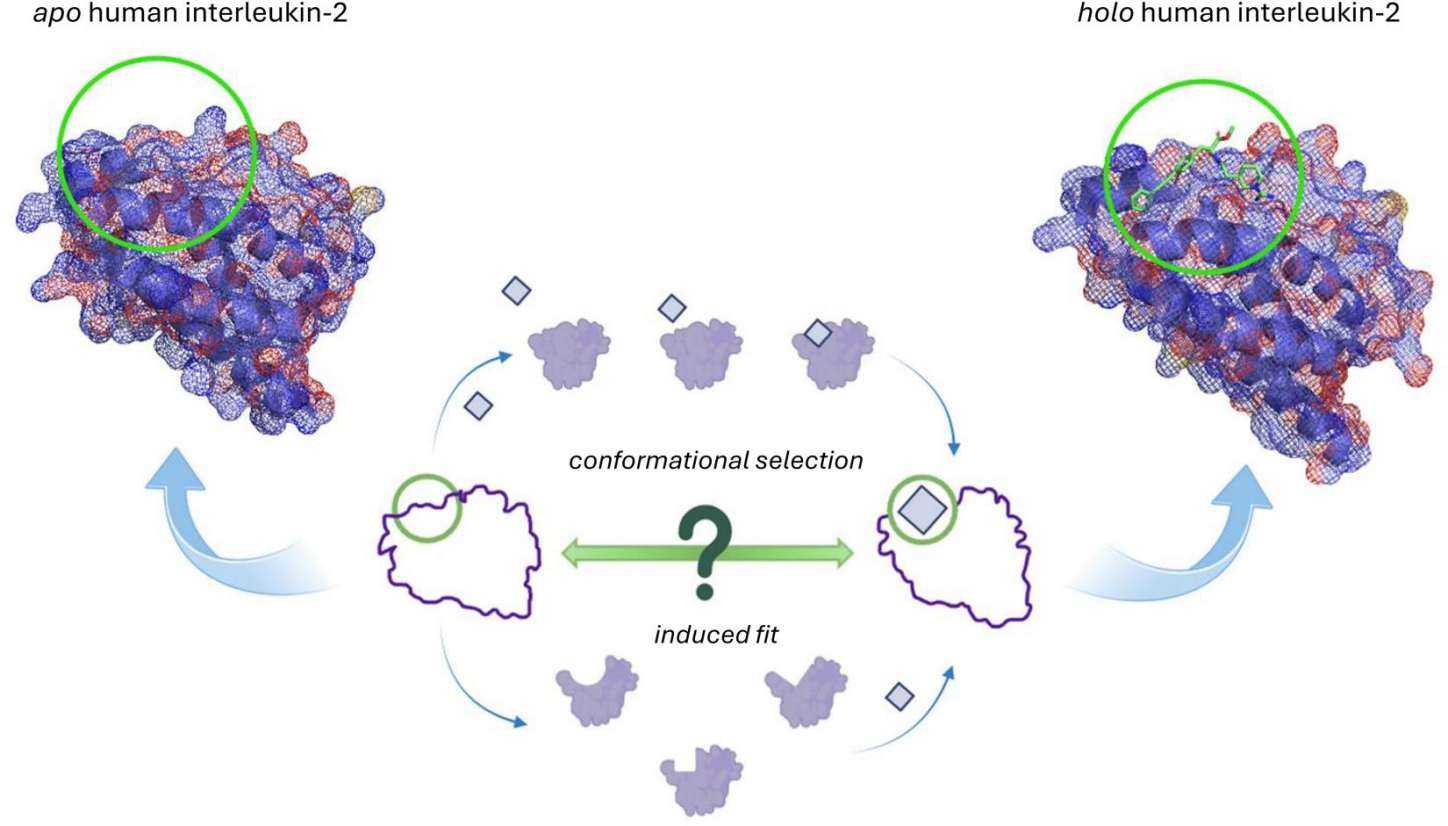
Cryptic binding site formation in human interleukin-2 (IL-2) (PDB code 1m47 corresponding to the apo protein and 1m48 corresponding to the holo protein) (Arkin *et al.* 2003). In the presence of the ligand, a druggable pocket is formed to accommodate the ligand. The mechanism of cryptic binding site formation is unknown, but it is theorized to either be created via induced fit or conformational selection. In conformational selection, the ligand stabilizes specific conformations that are also accessible in the unbound state, while during induced fit, the ligand causes the target to explore different conformations that are inaccessible in the unbound form.

In recent years, many computational advances have contributed to the enrichment of structure-based drug design, ranging from quantum mechanical calculations (Borbulevych *et al.* 2021) to deep learning methods (Özçelik *et al.* 2023). Furthermore, there has been an increased focus on protein-ligand sites, which can be broadly classified as active and allosteric (see Guarnera and Berezovsky 2016, for more details). There are many known cases where protein-ligand binding sites are inaccessible in their *apo* forms. However, in the presence of a bound ligand (*holo* form), they can become accessible. These ligand binding sites, which are not visible in the *apo* form but exist in the *holo* form, are referred to as cryptic sites or cryptic binding sites (Fig. 1) (Cimermancic *et al.* 2016, Vajda *et al.* 2018).

Proteins are highly dynamic structures that undergo conformational changes, which can cause accessibility challenges in seemingly well-defined catalytic binding sites and allosteric sites. Allosteric sites can be located near the active site or in a remote location and be affected by the dynamic nature of proteins in both cases. Many factors, including the lack of success of structure-based drug design for certain significant proteins, cofactor competition at the active site, and the inability of a cavity to bind a drug-size molecule, have led to increased interest in the study of cryptic binding sites (Hopkins and Groom 2002, Chio *et al.* 2015, Oleinikovas *et al.* 2016, Hart *et al.* 2017, Lu *et al.* 2018). A better understanding and identification of these sites opens up novel opportunities for structure-based drug design by allowing the targeting of proteins previously considered undruggable (Lazou *et al.* 2024, Bemelmans *et al.* 2025). Furthermore, targeting cryptic binding sites is advantageous as they can be targeted with increased specificity. Additionally, cryptic binding sites allow for distinct pharmacological profiles, ligand optimization, and they can serve as viable alternatives when the main active site of a protein is difficult to target (Hardy and Wells 2004, Arkin and Whitty 2009, Lu *et al.* 2018). However, the question regarding the usefulness of cryptic sites for drug discovery has been raised in the past. Hajduk *et al.* postulated that a protein could be designated druggable if (a) a ligand can bind to it with an affinity high enough for the modulation of biological activity and (b) it complies with the Rule of Five (Ro5) or the binding ligands are macrocycles, peptidomimetics or other compounds larger than 500 Da (Lipinski *et al.* 2001, Hajduk *et al.* 2005, Doak and Kihlberg 2016, Doak *et al.* 2016).

There are several reports where the druggability of cryptic binding sites was investigated using a wide range of computational methods. Kazakov *et al.* and Beglov *et al.* used FTMap to investigate cryptic binding sites based on the number of distributed organic probe clusters and suggested a cryptic binding site is druggable when it can bind 16 or more probe clusters (Kozakov *et al.* 2015a, 2015b). Horn and Shoichet (2004) reported a cryptic site serendipitously identified in a TEM-1 β-lactamase, which was found during enzyme inhibition and crystallization experiments with two different ligands.

Cimermancic *et al.* (2016) developed the CryptoSite program to identify and study cryptic binding sites using a benchmark set of 93 unbound-bound protein pairs. They successfully classified the site in each pair to be cryptic or non-cryptic based on the sequence, structure, several dynamic attributes, and machine learning based predictions. Importantly, a range limited sample PDB dataset for testing tools for cryptic binding sites has already been published (Cimermancic *et al.* 2016, Ge *et al.* 2024).

Systematic searches using MD-based methods for the detection of cryptic binding sites in proteins were reported later. It began with the use of small molecule and fragment library screening for cryptic binding site detection in several different proteins, including CDK2 (Hardy and Wells 2004, Ludlow *et al.* 2015). Additionally, the use of antibodies and site directed tethering was implemented to identify inhibitors of NDH-2 (Erlanson *et al.* 2004, Hardy and Wells 2004, Ludlow *et al.* 2015). However, in both cases, a large experimental set-up was required with a limited chance of success. This resulted in slow progress in this area until recently when better computational methods and hardware support led Bowman and Geissler to report a Markov State Model (MSM) built on a large MD simulation that identified a prospective cryptic site. They reported a known cryptic site in the TEM β-lactamase to be partially open for 53% of the simulation time (Bowman *et al.* 2015).

## 2 Overview of computational tools to detect cryptic binding modes

**Figure 2.**
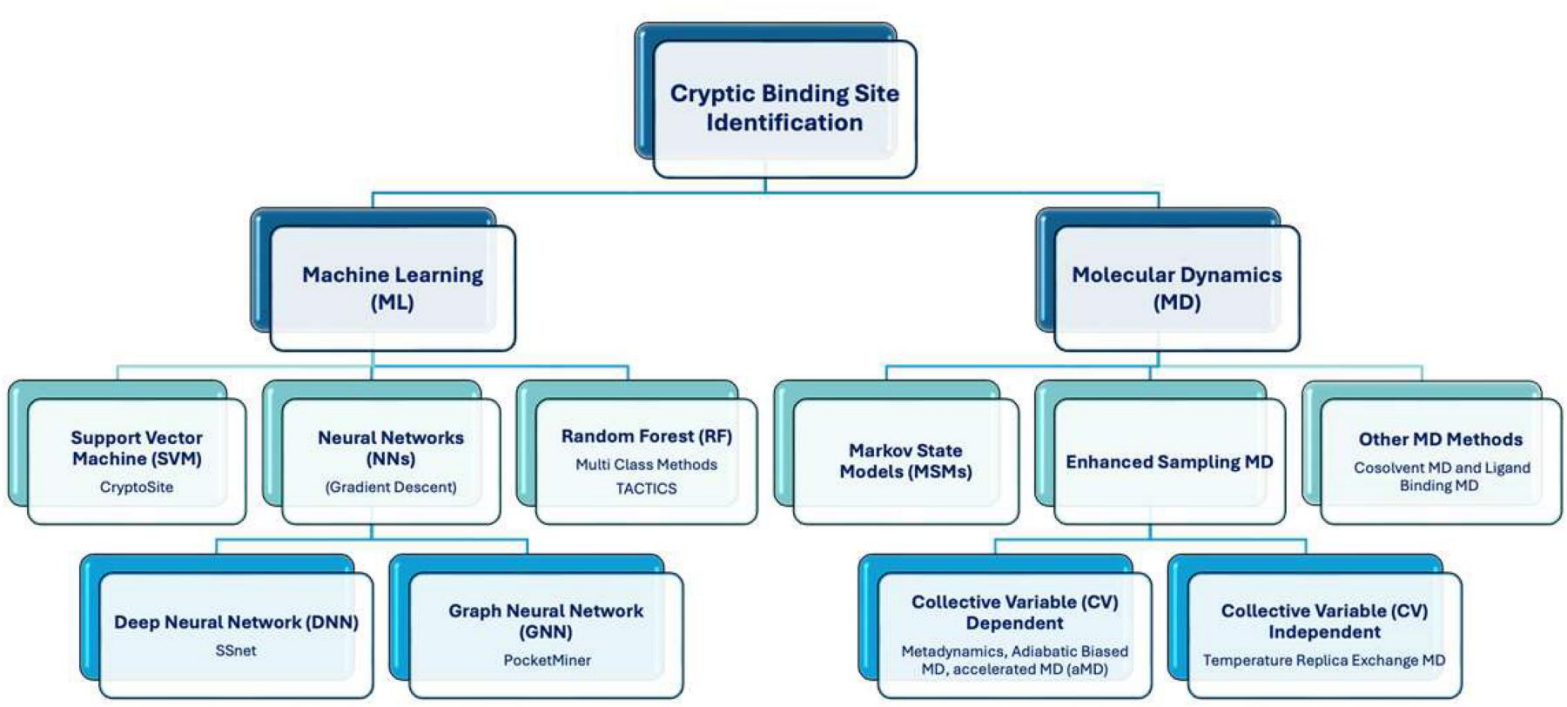
Schematic representation of computational methods specifically used in the identification of cryptic binding sites. These are broadly classified into MD methods and ML methods. CryptoSite is available online and it takes 1–2 days to obtain a prediction based on a PDB file (Cimermancic *et al.* 2016). The SSnet script can be found on GitHub (Verma *et al.* 2021). PocketMiner is available online and prediction can be made within minutes (Meller *et al.* 2023). TACTICS can be found on GitHub, a prediction can be made within an hour (AMD R7, 2700X with 8 cores and 16 threads, 16 GB RAM) (Evans *et al.* 2021). Calculations times for MD methods vary widely depending on computer resources and simulations times. GROMACS was used to perform 1000 simulations using a Markov State Model (MSM) (Bowman *et al.* 2015) and aggregated to 81 μs on a distributed compute system Folding@Home (Bowman *et al.* 2015). CV dependent simulations (AMBER18, pmemd-CUDA) were conducted using GPU-accelerated hardware with both unrestrained classical MD and accelerated MD (aMD). Ten independent classical and accelerated MD simulations of 100 ns were performed resulting in 1 μs of aggregated sampling per probe (Smith and Carlson 2021) Metadynamics (NAMD 2.12) was used to run 100 ns of unbiased MD simulations (Benabderrahmane *et al.* 2021). Adiabatic biased MD (Desmond, GPU) using a NVIDIA GTX 1080 graphic card on a 4-GPU computer was performed with three proteins for 20 ns each (Sun *et al.* 2020). The replica exchange method SWISH was performed with 20 replicas (water-benzene) with each running for 0.3 μs (Comitani and Gervasio 2018). Mixed Solvent MD was run on Desmond GPU and NVIDIA GTX 780 100 ns for each co-solvent system (Kimura *et al.* 2017).

Several new methods and algorithms are being used for the detection and analysis of cryptic sites in target protein molecules. These can be broadly divided in two classes: physics-based methods, ie, molecular dynamics (MD) and machine learning (ML) methods, respectively (as shown in Fig. 2 and summarized in Table 1). Here, we will explore the MD and ML based methods that have been reported recently for the identification of cryptic binding sites.

**Table 1.** Summary of MD simulation and ML based computational methods specifically used in the identification of cryptic binding sites.

	Method		Key features	Advantages	Limitations	Examples	References
**MDS Methods**	**Markov State Models (MSMs)**		Utilizes multiple classical MD simulation trajectories of a protein system.	Integrates different independent simulations, capturing long timescales.	Computationally expensive.	TEM-1 β-lactamase, MetAP-II, D3R	Bowman *et al.* 2015 Rubina *et al.* 2024 Ferruz *et al.* 2018 Husic and Pande 2018
	**Enhanced Sampling MD (ESMD)**	**Collective Variable (CV) dependent**	Dependent on predefined collective variables.	Improves issues with sampling that can lead to identifying pocket formation incorrectly.	Trapping of protein in local minima, failure to identify high energy states relies on aforementioned information of cryptic pocket location.	FGFR1, McI-1	Kuzmanic *et al.* 2020 Invernizzi and Parrinello 2022 Benabderrahmane *et al.* 2021
		**Collective Variable (CV) independent**	Independent of collective variables such as temperature.	Highly efficient for systems with slow and rare events where a cryptic site is identified due to protein unfolding.	High computational power required and difficulty during calculation set-up due to target specificity.	IL2, PLK1, NPC2, LfrR, p38α, hPNMT, DYRK1A kinase	Kuzmanic *et al.* 2020 Kii *et al.* 2016 Invernizzi and Parrinello 2022 Rizzi *et al.* 2023
	**Cosolvent MD**		The use of cosolvents, small organic molecules, to identify cryptic binding sites.	Does not rely on a priori knowledge, uses target independent parametrization.	Requires careful selection of co-solvents and high computing power.	Ricin, ALBP, AR, BACE-1, Hsp90	Smith and Carlson 2021 Martinez-Rosell *et al.* 2020 Lexa *et al.* 2014 Tze-Yang Ng and Tan 2022
**ML Methods**	**Support Vector Machine (SVM)** *CryptoSite*		Focuses on identifying amino acid residue changes during ligand binding to identify cryptic binding sites.	Designed specifically for cryptic binding site detection.	Experiences false-positive predictions and some cryptic sites are harder to find.	PTP1B	Cimermancic *et al.* 2016
	**Neural Networks** (NNs)	**Graph NN (GNN)** *PocketMiner*	Focuses on cryptic binding pocket opening events to identify cryptic binding sites.	Good at discriminating between residues that form cryptic pockets and ones that do not.	Reliant on MD simulations, use of PocketMiner is recommended with MD simulations after.	WNT2, PIM2	Meller *et al.* 2023
		**Deep NN (DNN)** *Ssnet*	Utilizes the grad-CAM analysis and focuses on backbone structural information of proteins.	Does not exhibit biases in physiochemical properties.	Blindness to conformation, resulting in lower ability to differentiate between binding sites.	PKA, TK	Verma *et al.* 2021
	**Random Forest (RF)** *TACTICS*		Trained on a reconstructed CryptoSite database set and uses ConCavity.	Can determine the druggability of a binding site using fragment docking.	Assumes all cryptic binding sites are closed, which is not always the case.	SARS-CoV-2 nsp5, SARS-CoV-2 MTase, ArCP	Evans *et al.* 2021

Some examples and references are included.

### 2.1 MD simulations methods

**Figure 3.**
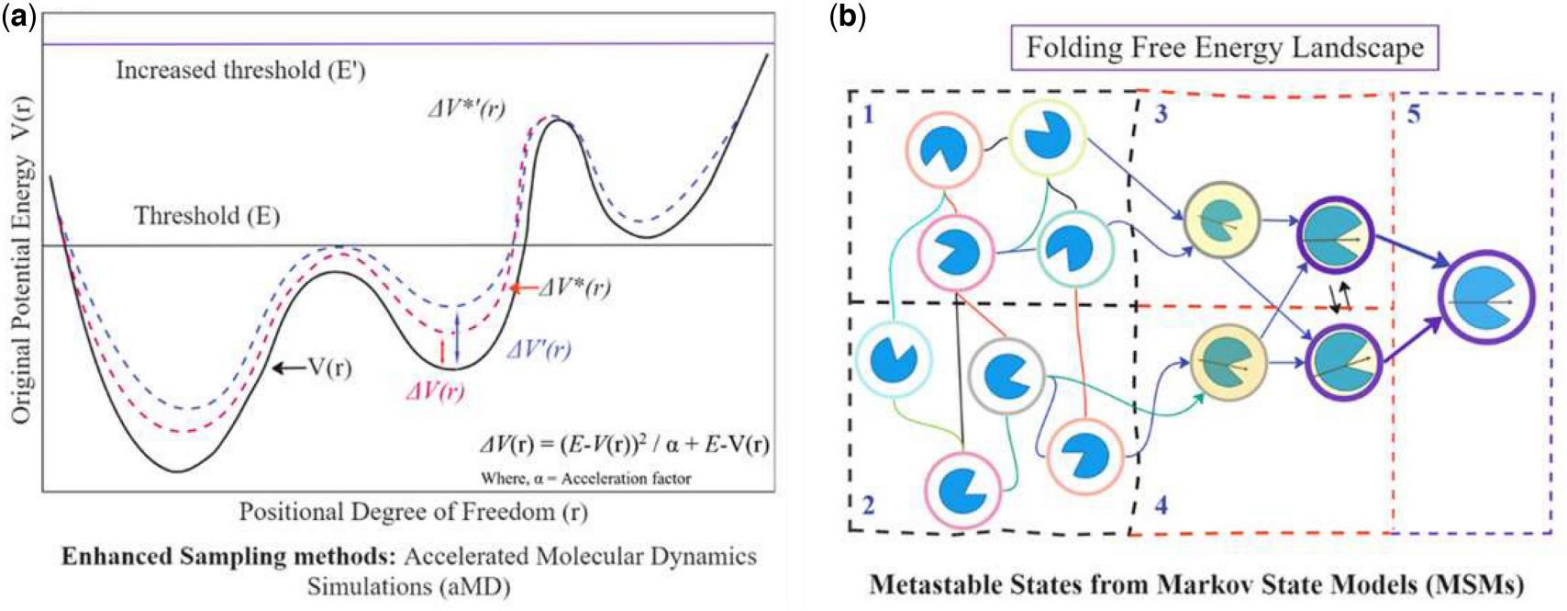
Molecular dynamics simulations for the identification of cryptic binding pockets: (a) non-classical MD and enhanced sampling techniques like accelerated MD, umbrella sampling, metadynamics, parallel tempering, and replica exchange methods have been successful in the past. However, they require some prior knowledge of the location of the binding sites. (b) MSMs use multiple classical MD simulation trajectories of a protein system. MSMs cluster similar conformations from the system to build microstates, which are used to estimate transition probabilities.

The work on cryptic binding sites gained traction during the early years of the 21st century when experimental studies revealed the presence of cryptic sites along with allosteric binding sites (Grimsley *et al.* 2005). Several MD simulation methods have been explored for the detection of cryptic binding sites, including MD simulations in combination with experimental studies, enhanced sampling methods, cosolvent MD simulations, replica exchange methods, and MSMs (Fig. 3).

The combination of experimental structure determination with explicit solvent MD simulations led to the successful sampling of MAP kinase p38’s cryptic binding site (Pargellis *et al.* 2002). In this kinase, a 10 Å movement of a phenylalanine side chain created a new binding site, which was exploited by a novel inhibitor, BIRB796. Consequently, the authors show that this cryptic binding site can be successfully identified starting with the *apo* structure by explicit solvent MD simulation methods (Frembgen-Kesner and Elcock 2006). Furthermore, many cryptic binding sites were identified while working on allosteric site modulations. While targeting the glutamate racemase enzyme from *Bacillus anthracis*, Whalen *et al.* (2011) characterized a novel cryptic binding site using polyacrylamide gel electrophoresis, MD simulations, free energy, and p*K*_a_ calculations. Cryptic sites are often less hydrophobic compared to other binding sites and can be stabilized by a mix of water and small probes. These can be miscible with water, including acetic acid, isopropanol, resorcinol, or hydrophobic, including benzene. While using probes for detection, caution is required in the estimation of the repulsive potential, which might lead to the collapse of the system by clustering of the probes (Seco *et al.* 2009, Bakan *et al.* 2012, Kimura *et al.* 2017, Schmidt *et al.* 2019, Yanagisawa *et al.* 2021). This method, using hydrophobic ring probes, was successful in the identification of a cryptic binding pocket in TEM-1 β-lactamase. The most recent successful example of cryptic binding site identification was reported by the Majumdar group, where they reported application of classical MD simulation to identify a cryptic binding pocket in a GPCR, the Cannabinoid receptor type 1 (CB1). This led to the design and optimization of the positively charged derivatives of CB1 agonist MDMB-Fubinaca allowing access to the target binding residues through opening of cryptic pocket (Rangari *et al.* 2025).

The application of mixed-solvent MD still requires substantial sampling of the system. At times, ineffective sampling can fail to identify the formation of a pocket (Kuzmanic *et al.* 2020). To overcome these challenges, several methods can be used, including collective variable (CV) dependent enhanced sampling methods, which require enhanced sampling of configurational space and dimensionality reductions methods. CV dependent methods include metadynamics used to estimate free energies among other state functions of a given system (Schmidt *et al.* 2008, Barducci *et al.* 2011, Benabderrahmane *et al.* 2021), umbrella sampling (Torrie and Valleau 1977), accelerated MD (Smith and Carlson 2021), adiabatic biased MD (Sun *et al.* 2020), and steered MD (Gullingsrud *et al.* 1999). CV dependent enhanced sampling methods were successfully used in the investigation of the SSR128129E (SSR) binding mechanism to the receptor tyrosine kinase (RTK) and the development of newer inhibitors for fibroblast growth factor receptor 1 (FGFR1) (Bono *et al.* 2013). This is particularly significant due to the key role of RTK in signalling; its dysregulation can lead to cancer and many other human diseases. Therefore, RTK is a validated drug target and understanding it better can potentially improve current cancer therapies (Du and Lovly 2018). Despite these successes, these methods also have their disadvantages. One of the disadvantages of the CV method is its reliance on prior knowledge of the location of the binding site in the protein and its dependency on geometric criteria, which can make describing the site with parameters complex (Kuzmanic *et al.* 2020). One of the approaches to try and overcome this problem is by quantifying the pocket’s ability to accommodate a drug-like molecule. This can be achieved using a large 3D cubic grid that can be overlapped on the region of interest in the target protein and used to assess the druggability (Kuzmanic *et al.* 2020). Other limitations of enhanced sampling methods include multi-microsecond simulations and the necessity of large volumes of sampling, which require GPU based calculations and thus higher costs. However, overall, enhanced sampling methods have proven to be successful in identifying cryptic binding sites and have been reported widely.

Enhanced sampling methods independent of the collective variable, such as temperature replica exchange MD, have been reported to sample cryptic pockets (Sugita and Okamoto 1999). Oleinikovas and others explored the hydrophobic nature of the cryptic binding pockets by using a method that samples water interactions through scaled Hamiltonians (SWISH) (Comitani and Gervasio 2018). In SWISH, the noncovalent interactions between water molecules and non-polar carbons and sulphurs are scaled. This method can be used alone or in combination with other enhanced sampling methods and was evaluated and found useful in the detection of binding pockets in β-lactamase, IL2, PLK1, NPC2, LfrR, p38α, and hPnMT, with a shorter sampling time of 1 µs and 6–8 replicas. Where protein unfolding triggers the revelation of a cryptic binding site, methods independent of collective variables are highly efficient. An example of this was reported by Hagiwara *et al.*, where the unfolding process led to the identification of a cryptic binding site in the DYRK1A kinase (Kii *et al.* 2016). Several other methods with low CV dependence have also been reported, allowing suboptimal CVs to induce fast convergence and exploration of the enhanced sampling of the system. An example of a method like this is On-the-fly Probability Enhanced Sampling (OPES) and its derivatives oneOPES and OPESexplore (Invernizzi and Parrinello 2022, Rizzi *et al.* 2023).

The MSMs (Bowman *et al.* 2015) method for the detection of cryptic binding sites is also being utilized. This MD simulation method was originally developed to gain insight into protein folding, but is also applicable in a wider range of protein studies (Ferruz *et al.* 2018, Husic and Pande 2018, Rubina *et al.* 2024). MSMs were successfully applied for the detection of cryptic binding sites in the apo TEM-1 β-lactamase enzyme (Bowman *et al.* 2015). One of the main disadvantages of using MSMs is their use of multiple microsecond simulations, leading to high GPU costs. Other MD simulation-based approaches like cosolvent MD (Smith and Carlson 2021) and ligand binding MD methods are also useful in the detection of cryptic binding sites (Kuzmanic *et al.* 2020). Specifically, both have been essential for the identification of cryptic binding sites in challenging targets like GPCRs. Cosolvent MD and ligand binding MD make use of classical and enhanced sampling methods with a variety of solvents, which act as probes to identify hidden pockets. One of the main disadvantages of these methods is the requirement of high computing power and difficulty in setting up calculations which may vary from target to target (Lexa *et al.* 2014, Martinez-Rosell *et al.* 2020, Smith and Carlson 2021, Tze-Yang Ng and Tan 2022). Another example of a successful application of enhanced sampling and MSMs was the identification of cryptic binding sites in the KRAS receptor, leading to the discovery of a potent noncovalent and selective KRAS inhibitor, MRTX1133 (Porter *et al.* 2019).

### 2.2 Classical machine-learning based methods

Compared to MD simulations, which are computationally expensive, ML methods can facilitate the screening of a large number of targets more cost- and time-efficiently. Several ML methods exist for the prediction of active binding sites, including PUResNet, DeepSite, P2Rank, Siteradar, and many more (Jiménez *et al.* 2017, Krivák and Hoksza 2018, Kandel *et al.* 2021, Evteev *et al.* 2023). However, these are not specifically designed to uncover cryptic binding sites, which are usually more challenging to predict. ML methods summarized below are specific to the identification of cryptic binding sites in a variety of protein structures.

**Figure 4.**
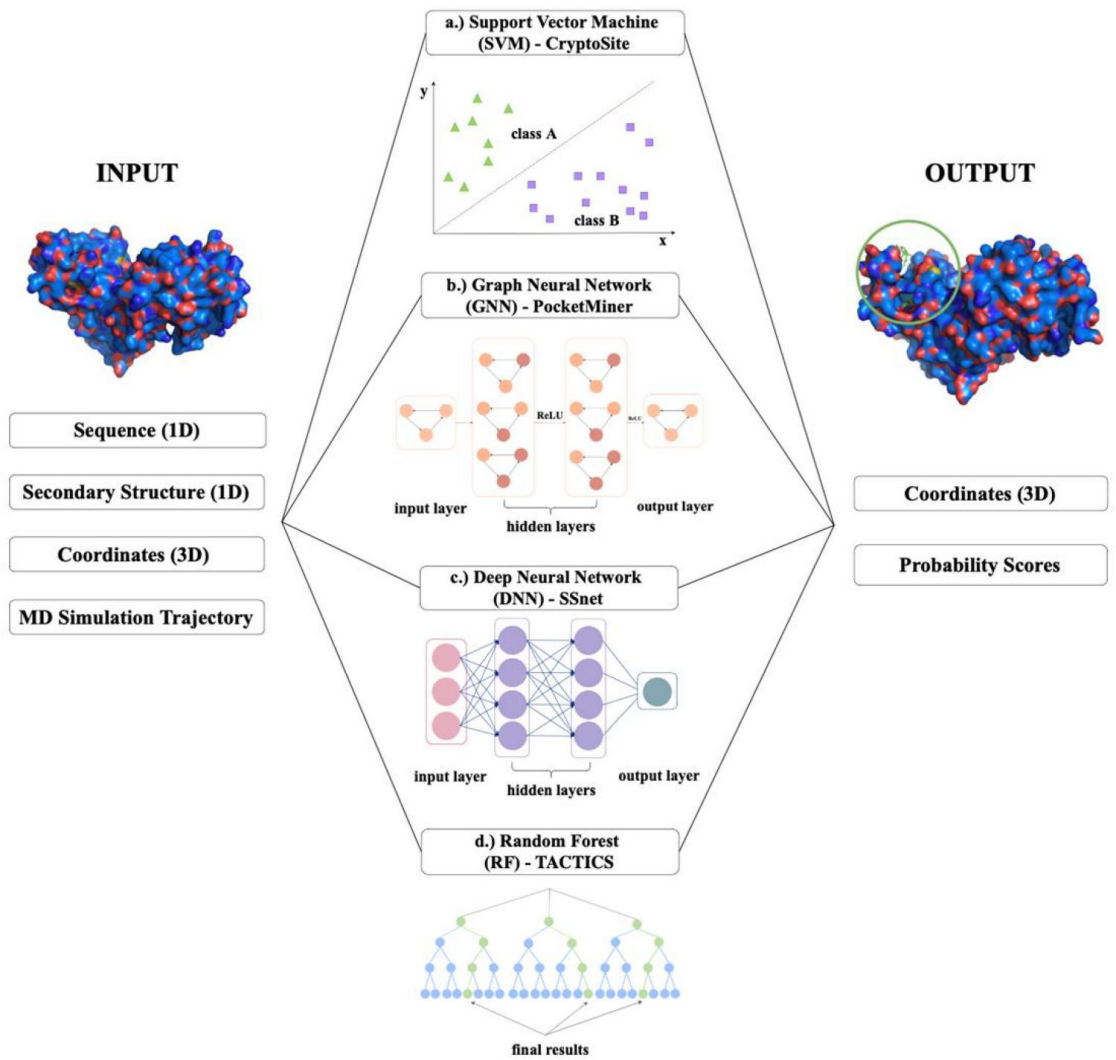
Schematic illustration of the several types of ML approaches for the determination of cryptic binding sites in protein structures. On the left, the apo p38 MAP kinase (PDB code: 5uoj) (Wang *et al.* 1997), and on the right, the same protein bound to two molecules of octyl beta-D-glucopyranoside (PDB code: 2npq) (Diskin *et al.* 2008) are shown as an example of cryptic binding site presence. (a) SVM, a supervised machine learning algorithm, is primarily used for classification tasks and utilizes a quadratic kernel function. (b) GNNs are neural networks designed to operate on graph data, data structures containing nodes (represented by circles) and edges. Node features include backbone dihedral angles, while an example of an edge feature is a unit vector between residue alpha-carbons. (c) DNNs consist of multiple layers of non-linear operations (hidden layers). In SSnet, curvature and torsion data are fed into the neural network. (d) The random forest algorithm combines the output of multiple decision trees to create a single result and is, therefore, extensively also used for classification tasks.

One of the most commonly cited machine learning (ML) methods, CryptoSite (Cimermancic *et al.* 2016), was created by Cimermancic *et al.* in 2016. It is a supervised machine-learning algorithm that predicts ligand-binding cryptic pockets based on a protein input (Fig. 4a). The method defines a cryptic site as a site that forms a pocket in the *holo* structure but is absent in the *apo* structure. Its premise is based on the analysis of 84 known examples of cryptic binding sites, 92 binding sites, and 705 concave surface patches obtained from the Protein Data Bank (PDB) (Berman *et al.* 2000) and the “Mother of All Databases” (MOAD) (Hu *et al.* 2005) database. Similarities between the structure, sequence, and dynamics of the three different forms inform the machine-learning algorithm. CryptoSite utilizes the SVM (Support Vector Machine) algorithm in conjunction with the quadratic kernel function and scaling; the algorithm was chosen out of 11 tested algorithms due to its most accurate performance, resulting in a predictive model, assessed using both the training set and an independent test set. Consequently, CryptoSite is able to classify amino acid residues as participating in the formation of a cryptic site or not. Cimermancic *et al.* utilized this method to predict the cryptic site of a protein involved in the insulin signalling pathway, tyrosine phosphatase 1B (PTP1B). Performing a cryptic binding site prediction using CryptoSite usually takes 1–2 days on the web server. Hence, compared to MD simulations used for cryptic site discovery, CryptoSite, in general, provides a more time-efficient and cost-effective alternative.

### 2.3 Advanced ML methods

PocketMiner (Meller *et al.* 2023) is a graph neural network (GNN) designed to predict cryptic pocket opening events (Fig. 4b), in contrast to CryptoSite, which focuses on identifying the amino acid residues that undergo transitions during ligand binding. PocketMiner can use a higher number of training examples, which leads to higher model accuracy. PocketMiner’s approach leads to the creation of a model that exhibits an improved ROC value of 0.87 and a > 1000-fold increase in speed when compared to CryptoSite. PocketMiner is, however, still somewhat reliant on MD simulations as these provide the training data used for training models. The tool is based on a dataset of 37 proteins, and 2400 independent MD simulations (40 ns window) were used to train a geometric vector perceptron graph neural network (GVP-GNN) and a 3D convolutional neural network (3D-CNN). Between the two, GVP-GNN performs better with fewer false-positive predictions. Cryptic pocket opening evaluation is based on the pocket volume change, which was calculated using the LIGSITE algorithm (Hendlich *et al.* 1997) (created by Hendlich *et al.*) and the maximum Fpocket druggability scores. The method was applied across the human proteome to further test its performance, and novel cryptic sites were discovered in more than half of the proteins. This discovery displays the link between cryptic site prediction and expanding the druggable proteome for better drug design (Colombo 2023).

SSnet (Karki *et al.* 2021) is a DNN (deep neural network) framework that uses the backbone structural information of proteins [torsion (τ) and curvature (*K*) of the backbone, a secondary structure description also used in the ab initio structure determination by small-angle X-scattering technique] (Prior *et al.* 2020), to determine protein-ligand interactions (PLIs), making it useful for the prediction of cryptic binding sites (Fig. 4c). The grad-CAM (gradient-weighted Class Activation Mapping) analysis incorporated into the method plays a key role in elucidating which areas of the protein are more relevant to the prediction than others. Furthermore, when used with SSnet, it validated SSnet’s ability to accurately identify cryptic sites without prior knowledge of their regulatory roles. SSnet was tested on three different proteins in the CryptoSite set, and it was able to successfully predict their cryptic binding sites, suggesting it can be useful for the prediction of cryptic binding sites in the future. However, overall, one of SSnet’s limitations is its blindness to conformation. A similar limitation can also be observed when generating ligands via deep learning methods (Baillif *et al.* 2023, Malbranke *et al.* 2023). Conformation blindness is a potential issue because mutations may result in the same fold but induce a different interaction, which SSnet would not be able to differentiate. It should, therefore, be followed by further docking experiments and simulations to validate its predictions, giving it a disadvantage.

TACTICS (Evans *et al.* 2021) stands for ‘*T*rajectory-based *A*nalysis of *C*onformations *T*o *I*dentify *C*ryptic *S*ites’ (Fig. 4d) and similarly to the previously mentioned methods, it was developed to identify druggable binding sites. The TACTICS algorithm analyses a select group of frames in a MD simulation using a random forest ML model and selects potentially bindable residues based on protein motion and geometry. The model is trained on a reconstructed version of the CryptoSite database and determines the druggability of each amino acid residue in a specific protein conformation. One of the features of TACTICS is a pocket detection algorithm called ConCavity. ConCavity considers the geometry of the protein structure and the sequence conservation of each residue; the latter not being utilized in TACTICS yet. TACTICS was assessed and correctly identified ligand binding sites in SARS-CoV-2 proteins and predicted several new binding sites in the SARS-CoV-2 2’-O RNA methyltransferase and the *Yersinia pestis* aryl carrier protein. To eradicate the issue of certain proteins being crystallized more than others and having more crystallographic structures available, a fixed number of 50 *apo* structures per 1 *holo* structure was used. In addition to predicting cryptic binding sites, TACTICS also determines the druggability of a binding site via fragment docking. However, one of its shortcomings is that the procedure assumes that in all *apo* structures, cryptic binding sites are closed or unbindable. However, this might not always be the case; in some *apo* structures, the cryptic binding site can be open or partially open even without the presence of the ligand. In 2021, Wenjun Zheng published a paper mentioning a method based on normal modes guided conformational sampling. This method obtained higher FPR and TPR values compared to CryptoSite and was trained on the same dataset for direct comparison. The method combines and cross-validates three ML protocols (random forest, neural net, and LASSO regularization) for its performance. Through this, it is able to reach even faster prediction times of around 1–2 hours for an average-size protein (Zheng 2021).

## 3 Comparison of MD and ML methods

**Figure 5.**
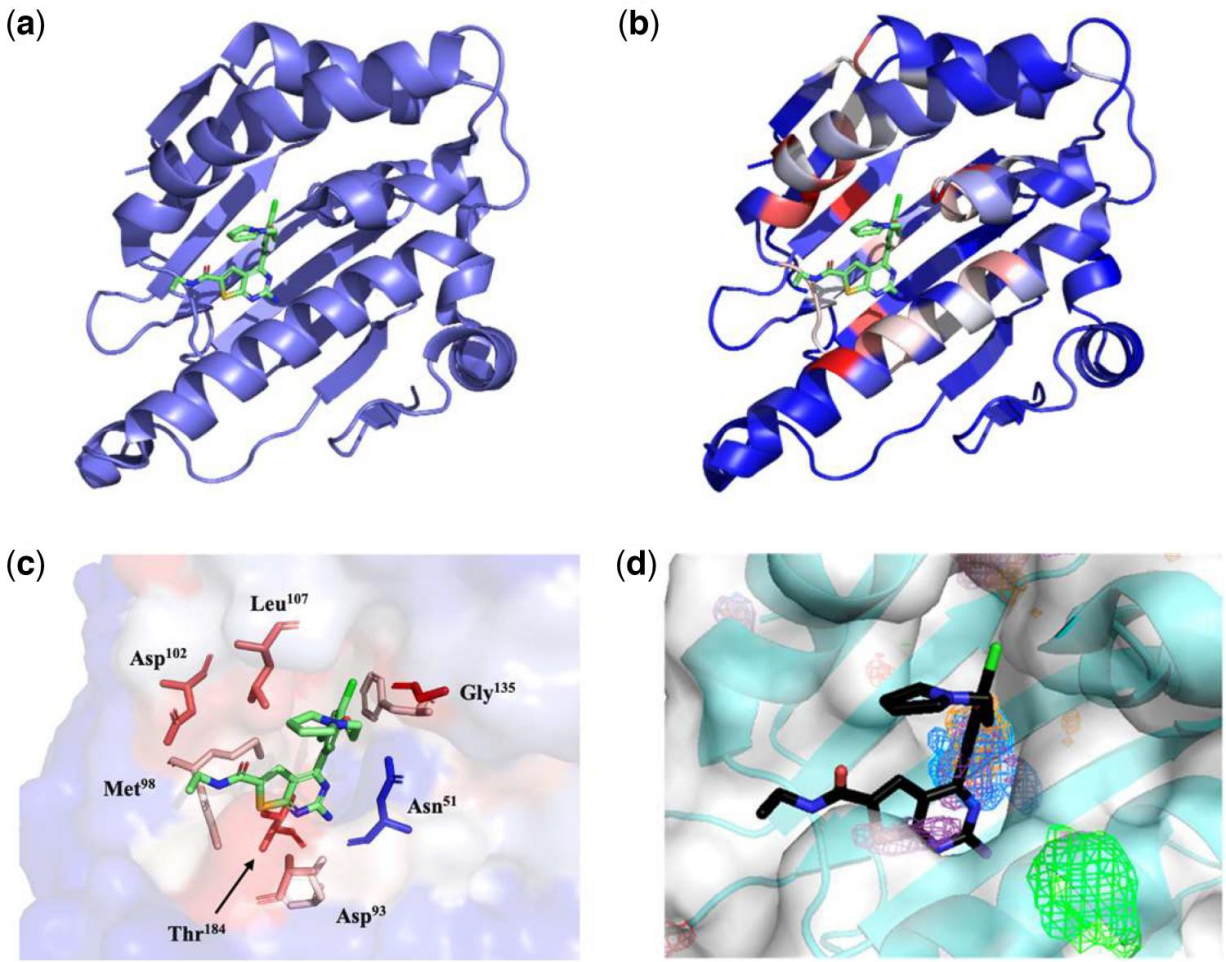
(a) Hsp90 (PDB entry 2wi7) bound to an inhibitor shown in green. (b) Hsp90 (PDB entry: 2wi7) coloured based on CryptoSite scoring. Shades of red indicate a higher probability of amino acid residue contribution to a cryptic binding site. Shades of blue indicate a lower probability of an amino acid residue participating in a cryptic binding site. (c) Close-up of the Hsp90 cryptic binding site, the protein is represented as a surface with some key residues highlighted. (d) Binding site of Hsp90 as determined by standard MixMD with probes shown in mesh (Smith and Carlson 2021).

To evaluate CryptoSite’s (http://salilab.org/cryptosite) ability to correctly predict a cryptic binding site we compared the program with previously established MD simulation (see Supplementary Material, available as supplementary data at *Bioinformatics Advances* online). These examples were selected from publications mentioned in the MD—method section of Table 1 and included a variety of different protein systems. PDB files for each protein entry were selected and default parameters were used to perform CryptoSite evaluation. In most cases, CryptoSite successfully identified the known cryptic binding sites. Amino acid residues contributing to cryptic binding site formation were scored and colour-coded with red amino acid residues indicating higher probability of cryptic binding site contribution, and blue amino acid residues indicating the opposite. In one of the examples, Hsp90 (PDB entry: 2wi7) a molecular chaperone protein was coloured according to CryptoSite’s scoring evaluation (Fig. 5). The accelerated MD simulation performed with 2wi7 was not able to identify the cryptic binding site in contrast to CryptoSite, which pinpointed the expected amino acid residues (Smith and Carlson 2021). Compared to standard MixMD, CryptoSite was seemingly more successful in identifying the key amino acid residues. The different residues comprising the cryptic binding site and their respective CryptoSite scores can be found in Table 2.

**Table 2.** Amino acid residue predicted to be part of a cryptic binding site as determined by CryptoSite.

Amino acid residue	CryptoSite value	Type of interaction
Gly^135^	0.33	Backbone
Asn^51^	0.03	Side chain
Asp^93^	0.20	Side chain
Thr^184^	0.28	Side chain
Met^98^	0.21	Side chain
Ala^55^	0.24	side chain
Asp^102^	0.27	Side chain
Leu^107^	0.27	Side chain
Phe^138^	0.20	Side chain
Gly^97^	0.19	Backbone

The type of interaction observed in the original PDB entry: 2wi7 and the CryptoSite value assigned to each residue is also included. Out of the amino acid residues contributing to cryptic binding in Hsp90, only Asn^51^ reached a CryptoSite value of < 0.1.

Across different cases, CryptoSite’s accuracy varied depending on the protein system; however, overall CryptoSite was able to detect and highlight cryptic binding site existence. Its primary limitation was the exclusion of certain amino acid residues contributing to the formation of the pocket in some cases.

## 4 Current challenges and future directions

In conclusion, several ML methods have been developed to identify cryptic binding sites that are significant for the further understanding of protein-ligand interactions and structure-based drug discovery. However, to date, there is limited evidence of any truly novel ligands being discovered as a result of cryptic binding site detection using one of the ML-methods summarized above. One of the biggest challenges of machine learning methods lies in their reliance on the experimentally determined structures and their known protein-ligand complexes. The key limitation remains the limited learning set of experimentally derived structures with validated cryptic binding sites. Consequently, purely ML-based methods often fail to identify useful binding poses (Buttenschoen *et al.* 2024).

It is obvious that not all possible binding sites in a protein have been experimentally determined and verified by bound ligands. This means that many locations can be labelled as non-binding sites even though they might be binding sites that have not been discovered yet or represented. Even more worrying is that the fact that a significant number of protein-ligand complexes in the PDB, with estimates of up to 10%, show limited or no electron density for the ligand and might require further validation as a community effort (Deller and Rupp 2015, Rupp *et al.* 2016, Pozharski *et al.* 2017). An excellent example of such community validation initiative is the assessment of SARS CoV-2 drug targets in the PDB by with the aim to provide an improved set of structures for structural biologists and modellers worldwide (Wlodawer *et al.* 2020, Jaskolski *et al.* 2021). Consequently, persisting errors and/or inaccuracies in the PDB have a major impact on the models constructed by machine learning as they are based on datasets originating from the PDB. However, it is also clear that with an ever-expanding dataset of validated protein-ligand structures, ML method will play an important role in the future, in particular when searching for new sites in certain protein target classes such as kinases where a large number of closely related structures are available (Umezawa and Kii 2021, Yang *et al.* 2023). Even though cryptic binding sites are inherently underrepresented, more and more examples of protein-ligand complexes with binding sites are being discovered, enriching our current datasets, which can be utilized for further training.

In the meantime, ML-methods working in parallel with physics-based model such as coarse grain (Kjølbye *et al.* 2022) and/or molecular dynamics simulations to postulate where potential cryptic binding sites are located may offer the greatest potential (Bemelmans *et al.* 2025). In the future, existing MD/ML methods for the prediction of active binding sites can be tweaked and trained on a wide range of different protein targets sets to make them more specific for the prediction of cryptic binding sites. This is already being done in approaches such as SiteMap that applies enhanced sampling molecular dynamics techniques. (Ge and Ganamet 2023). Finally, ML methods offer unique opportunities to combine not only experimental and computational data but also biochemistry and ultimately biomedical data. The integration of a wide range of diverse date offers the greatest potential for ML based methods for future drug discovery.

## Supplementary Material

vbaf156_Supplementary_Data

## Data Availability

The data underlying this article will be shared on reasonable request to the corresponding author.

## References

[vbaf156-B1] Arkin MR , Randal M, DeLano WL et al Binding of small molecules to an adaptive protein-protein interface. Proc Natl Acad Sci U S A 2003;100:1603–8. 12582206 10.1073/pnas.252756299 PMC149879

[vbaf156-B2] Arkin MR , Whitty A. The road less traveled: modulating signal transduction enzymes by inhibiting their protein–protein interactions. Curr Opin Chem Biol 2009;13:284–90. 19553156 10.1016/j.cbpa.2009.05.125

[vbaf156-B3] Baillif B , Cole J, Giangreco I et al Applying atomistic neural networks to bias conformer ensembles towards bioactive-like conformations. J Cheminform 2023;15:124–3. 38129933 10.1186/s13321-023-00794-w PMC10740246

[vbaf156-B4] Bakan A , Nevins N, Lakdawala AS et al Druggability assessment of allosteric proteins by dynamics simulations in the presence of probe molecules. J Chem Theory Comput 2012;8:2435–47. 22798729 10.1021/ct300117j PMC3392909

[vbaf156-B5] Barducci A , Bonomi M, Parrinello M et al Metadynamics. WIREs Comput Mol Sci 2011;1:826–43.

[vbaf156-B6] Bemelmans MP , Cournia Z, Damm-Ganamet KL et al Computational advances in discovering cryptic pockets for drug discovery. Curr Opin Struct Biol 2025;90:102975. 39778412 10.1016/j.sbi.2024.102975

[vbaf156-B7] Benabderrahmane M , Bureau R, Voisin-Chiret AS et al Cryptic pockets repository through pocket dynamics tracking and metadynamics on essential dynamics space: applications to Mcl-1. J Chem Inf Model 2021;61:5581–8. 34748701 10.1021/acs.jcim.1c00660

[vbaf156-B8] Berman HM , Westbrook J, Feng Z et al The protein data bank. Nucleic Acids Res 2000;28:235–42. 10592235 10.1093/nar/28.1.235 PMC102472

[vbaf156-B9] Bono F , De Smet F, Herbert C et al Inhibition of tumor angiogenesis and growth by a small-molecule multi-FGF receptor blocker with allosteric properties. Cancer Cell 2013;23:477–88. 23597562 10.1016/j.ccr.2013.02.019

[vbaf156-B10] Borbulevych OY , Martin RI, Westerhoff LM et al The critical role of QM/MM X-ray refinement and accurate tautomer/protomer determination in structure-based drug design. J Comput Aided Mol Des 2021;35:433–51. 33108589 10.1007/s10822-020-00354-6 PMC8018927

[vbaf156-B11] Bowman GR , Bolin ER, Hart KM et al Discovery of multiple hidden allosteric sites by combining Markov state models and experiments. Proc Natl Acad Sci U S A 2015;112:2734–9. 25730859 10.1073/pnas.1417811112 PMC4352775

[vbaf156-B12] Buttenschoen M , Morris GM, Deane CM et al PoseBusters: AI-based docking methods fail to generate physically valid poses or generalise to novel sequences. Chem Sci 2024;15:3130–9. 38425520 10.1039/d3sc04185a PMC10901501

[vbaf156-B13] Chio CM , Lim CS, Bishop AC et al Targeting a cryptic allosteric site for selective inhibition of the oncogenic protein tyrosine phosphatase Shp2. Biochemistry 2015;54:497–504. 25519989 10.1021/bi5013595 PMC4303306

[vbaf156-B14] Cimermancic P , Weinkam P, Rettenmaier TJ et al CryptoSite: expanding the druggable proteome by characterization and prediction of cryptic binding sites. J Mol Biol 2016;428:709–19. 26854760 10.1016/j.jmb.2016.01.029 PMC4794384

[vbaf156-B15] Colombo G. Computing allostery: from the understanding of biomolecular regulation and the discovery of cryptic sites to molecular design. Curr Opin Struct Biol 2023;83:102702. 37716095 10.1016/j.sbi.2023.102702

[vbaf156-B16] Comitani F , Gervasio FL. Exploring cryptic pockets formation in targets of pharmaceutical interest with SWISH. J Chem Theory Comput 2018;14:3321–31. 29768914 10.1021/acs.jctc.8b00263

[vbaf156-B17] Deller MC , Rupp B. Models of protein–ligand crystal structures: trust, but verify. J Comput Aided Mol Des 2015;29:817–36. 25665575 10.1007/s10822-015-9833-8 PMC4531100

[vbaf156-B18] Diskin R , Engelberg D, Livnah O et al A novel lipid binding site formed by the MAP kinase insert in p38α. J Mol Biol 2008;375:70–9. 17999933 10.1016/j.jmb.2007.09.002

[vbaf156-B19] Doak BC , Kihlberg J. Drug discovery beyond the rule of 5—opportunities and challenges. Expert Opin Drug Discov 2016;12:115–19. 10.1080/17460441.2017.1264385 27883294

[vbaf156-B20] Doak BC , Zheng J, Dobritzsch D et al How beyond rule of 5 drugs and clinical candidates bind to their targets. J Med Chem 2016;59:2312–27. 26457449 10.1021/acs.jmedchem.5b01286

[vbaf156-B21] Du Z , Lovly CM. Mechanisms of receptor tyrosine kinase activation in cancer. Mol Cancer 2018;17:58. 29455648 10.1186/s12943-018-0782-4 PMC5817791

[vbaf156-B22] Erlanson DA et al Tethering: fragment-based drug discovery. Annu Rev Biophys 2004;33:199–223. 10.1146/annurev.biophys.33.110502.140409 15139811

[vbaf156-B23] Evans DJ , Yovanno RA, Rahman S et al Finding druggable sites in proteins using TACTICS. J Chem Inf Model 2021;61:2897–910. 34096704 10.1021/acs.jcim.1c00204 PMC8631419

[vbaf156-B24] Evteev SA , Ereshchenko AV, Ivanenkov YA et al SiteRadar: utilizing graph machine learning for precise mapping of protein-ligand-binding sites. J Chem Inf Model 2023;63:1124–32. 36744300 10.1021/acs.jcim.2c01413

[vbaf156-B25] Ferruz N et al Dopamine D3 receptor antagonist reveals a cryptic pocket in aminergic GPCRs. Sci Rep 2018;8:1–10. 29343833 10.1038/s41598-018-19345-7 PMC5772633

[vbaf156-B26] Frembgen-Kesner T , Elcock AH. Computational sampling of a cryptic drug binding site in a protein receptor: explicit solvent molecular dynamics and inhibitor docking to p38 MAP kinase. J Mol Biol 2006;359:202–14. 16616932 10.1016/j.jmb.2006.03.021

[vbaf156-B27] Ge Y , Ganamet K. Using sitemap to aid in the identification of cryptic binding pockets. Biophys J 2023;122:142a.

[vbaf156-B28] Ge Y , Pande V, Seierstad MJ et al Exploring the application of SiteMap and site finder for focused cryptic pocket identification. J Phys Chem B 2024;128:6233–45. 38904218 10.1021/acs.jpcb.4c00664

[vbaf156-B29] Grimsley JK , Calamini B, Wild JR et al Structural and mutational studies of organophosphorus hydrolase reveal a cryptic and functional allosteric-binding site. Arch Biochem Biophys 2005;442:169–79. 16188223 10.1016/j.abb.2005.08.012

[vbaf156-B30] Guarnera E , Berezovsky IN. Allosteric sites: remote control in regulation of protein activity. Curr Opin Struct Biol 2016;37:1–8. 26562539 10.1016/j.sbi.2015.10.004

[vbaf156-B31] Gullingsrud JR , Braun R, Schulten K et al Reconstructing potentials of mean force through time series analysis of steered molecular dynamics simulations. J Comput Phys 1999;151:190–211.

[vbaf156-B32] Hajduk PJ , Huth JR, Fesik SW et al Druggability indices for protein targets derived from NMR-based screening data. J Med Chem 2005;48:2518–25. 15801841 10.1021/jm049131r

[vbaf156-B33] Hardy JA , Wells JA. Searching for new allosteric sites in enzymes. Curr Opin Struct Biol 2004;14:706–15. 15582395 10.1016/j.sbi.2004.10.009

[vbaf156-B34] Hart KM , Moeder KE, Ho CMW et al Designing small molecules to target cryptic pockets yields both positive and negative allosteric modulators. PLoS One 2017;12:e0178678. 28570708 10.1371/journal.pone.0178678 PMC5453556

[vbaf156-B35] Hendlich M , Rippmann F, Barnickel G et al LIGSITE: automatic and efficient detection of potential small molecule-binding sites in proteins. J Mol Graph Model 1997;15:359–63, 389. 9704298 10.1016/s1093-3263(98)00002-3

[vbaf156-B36] Hopkins AL , Groom CR. The druggable genome. Nat Rev Drug Discov 2002;1:727–30. 12209152 10.1038/nrd892

[vbaf156-B37] Horn JR , Shoichet BK. Allosteric inhibition through core disruption. J Mol Biol 2004;336:1283–91. 15037085 10.1016/j.jmb.2003.12.068

[vbaf156-B38] Hu L , Benson ML, Smith RD et al Binding MOAD (Mother of All Databases). Proteins 2005;60:333–40. 15971202 10.1002/prot.20512

[vbaf156-B39] Husic BE , Pande VS. Markov state models: from an art to a science. J Am Chem Soc 2018;140:2386–96. 29323881 10.1021/jacs.7b12191

[vbaf156-B40] Invernizzi M , Parrinello M. Exploration vs convergence speed in adaptive-bias enhanced sampling. J Chem Theory Comput 2022;18:3988–96. 35617155 10.1021/acs.jctc.2c00152 PMC9202311

[vbaf156-B41] Jaskolski M , Dauter Z, Shabalin IG et al Crystallographic models of SARS-CoV-2 3CL pro: in-depth assessment of structure quality and validation. IUCrJ 2021;8:238–56. 10.1107/S2052252521001159 PMC7924243 33708401

[vbaf156-B42] Jiménez J , Doerr S, Martínez-Rosell G et al DeepSite: protein-binding site predictor using 3D-convolutional neural networks. Bioinformatics 2017;33:3036–42. 28575181 10.1093/bioinformatics/btx350

[vbaf156-B43] Kandel J , Tayara H, Chong KT et al PUResNet: prediction of protein-ligand binding sites using deep residual neural network. J Cheminform 2021;13:65. 34496970 10.1186/s13321-021-00547-7 PMC8424938

[vbaf156-B44] Karki N et al SSnet: a deep learning approach for protein-ligand interaction prediction. Int J Mol Sci 2021;22:1–16. 10.3390/ijms22031392 PMC7869013 33573266

[vbaf156-B45] Kii I , Sumida Y, Goto T et al Selective inhibition of the kinase DYRK1A by targeting its folding process. Nat Commun 2016;7:11391–14. 27102360 10.1038/ncomms11391 PMC4844702

[vbaf156-B46] Kimura SR et al Deciphering cryptic binding sites on proteins by mixed-solvent molecular dynamics. ^J Chem Inform Model 2017;57:1388–401. 10.1021/acs.jcim.6b00623 28537745

[vbaf156-B47] Kjølbye LR , Pereira GP, Bartocci A et al Towards design of drugs and delivery systems with the Martinicoarse-grained model. QRB Discov 2022;3:e19. 37529288 10.1017/qrd.2022.16 PMC10392664

[vbaf156-B48] Kozakov D , Grove LE, Hall DR et al The FTMap family of web servers for determining and characterizing ligand-binding hot spots of proteins. Nat Protoc 2015a;10:733–55. 25855957 10.1038/nprot.2015.043 PMC4762777

[vbaf156-B49] Kozakov D , Hall DR, Napoleon RL et al New frontiers in druggability. J Med Chem 2015b;58:9063–88. 26230724 10.1021/acs.jmedchem.5b00586 PMC4762776

[vbaf156-B50] Krivák R , Hoksza D. P2Rank: machine learning based tool for rapid and accurate prediction of ligand binding sites from protein structure. J Cheminform 2018;10:39. 30109435 10.1186/s13321-018-0285-8 PMC6091426

[vbaf156-B51] Kuzmanic A , Bowman GR, Juarez-Jimenez J et al Investigating cryptic binding sites by molecular dynamics simulations. Acc Chem Res 2020;53:654–61. 32134250 10.1021/acs.accounts.9b00613 PMC7263906

[vbaf156-B52] Lazou M , Kozakov D, Joseph-McCarthy D et al Which cryptic sites are feasible drug targets? Drug Discov Today 2024;29:104197. 39368697 10.1016/j.drudis.2024.104197 PMC11568903

[vbaf156-B53] Lexa KW , Goh GB, Carlson HA et al Parameter choice matters: validating probe parameters for use in mixed-solvent simulations. J Chem Inf Model 2014;54:2190–9. 25058662 10.1021/ci400741u PMC4144759

[vbaf156-B54] Lipinski CA , Lombardo F, Dominy BW et al Experimental and computational approaches to estimate solubility and permeability in drug discovery and development settings. Adv Drug Deliv Rev 2001;46:3–26. 11259830 10.1016/s0169-409x(00)00129-0

[vbaf156-B55] Lu S , Ji M, Ni D et al Discovery of hidden allosteric sites as novel targets for allosteric drug design. Drug Discov Today 2018;23:359–65. 29030241 10.1016/j.drudis.2017.10.001

[vbaf156-B56] Ludlow RF , Verdonk ML, Saini HK et al Detection of secondary binding sites in proteins using fragment screening. Proc Natl Acad Sci U S A 2015;112:15910–5. 26655740 10.1073/pnas.1518946112 PMC4703025

[vbaf156-B57] Malbranke C , Bikard D, Cocco S et al Machine learning for evolutionary-based and physics-inspired protein design: current and future synergies. Curr Opin Struct Biol 2023;80:102571. 36947951 10.1016/j.sbi.2023.102571

[vbaf156-B58] Martinez-Rosell G , Lovera S, Sands ZA et al PlayMolecule CrypticScout: predicting protein cryptic sites using mixed-solvent molecular simulations. J Chem Inf Model 2020;60:2314–24. 32175736 10.1021/acs.jcim.9b01209

[vbaf156-B59] Meller A , Ward M, Borowsky J et al Predicting locations of cryptic pockets from single protein structures using the PocketMiner graph neural network. Nat Commun 2023;14:1177–15. 36859488 10.1038/s41467-023-36699-3 PMC9977097

[vbaf156-B60] Oleinikovas V , Saladino G, Cossins BP et al Understanding cryptic pocket formation in protein targets by enhanced sampling simulations. J Am Chem Soc 2016;138:14257–63. 27726386 10.1021/jacs.6b05425

[vbaf156-B61] Özçelik R , van Tilborg D, Jiménez-Luna J et al Structure-based drug discovery with deep learning. Chembiochem 2023;24:e202200776. 37014633 10.1002/cbic.202200776

[vbaf156-B62] Pargellis C , Tong L, Churchill L et al Inhibition of p38 MAP kinase by utilizing a novel allosteric binding site. Nat Struct Biol 2002;9:268–72. 11896401 10.1038/nsb770

[vbaf156-B63] Porter JR , Moeder KE, Sibbald CA et al Cooperative changes in solvent exposure identify cryptic pockets, switches, and allosteric coupling. Biophys J 2019;116:818–30. 30744991 10.1016/j.bpj.2018.11.3144 PMC6400826

[vbaf156-B64] Pozharski E et al Validation of protein–ligand crystal structure models: small molecule and peptide ligands. Methods Mol Biol 2017;1607:611–25. 28573591 10.1007/978-1-4939-7000-1_25

[vbaf156-B65] Prior C , Davies OR, Bruce D et al Obtaining tertiary protein structures by the ab initio interpretation of small angle X-ray scattering data. J Chem Theory Comput 2020;16:1985–2001. 32023061 10.1021/acs.jctc.9b01010 PMC7145352

[vbaf156-B66] Rangari VA et al A cryptic pocket in CB1 drives peripheral and functional selectivity. Nature 2025;640:265–73. 40044849 10.1038/s41586-025-08618-7 PMC11977287

[vbaf156-B67] Rizzi V , Aureli S, Ansari N et al OneOPES, a combined enhanced sampling method to rule them all. J Chem Theory Comput 2023;19:5731–42. 37603295 10.1021/acs.jctc.3c00254 PMC10500989

[vbaf156-B68] Rubina , Moin ST, Haider S, et al Identification of a cryptic pocket in methionine aminopeptidase-II using adaptive Bandit molecular dynamics simulations and Markov state models. ACS Omega 2024;9:28534–45. 38973915 10.1021/acsomega.4c02516 PMC11223136

[vbaf156-B69] Rupp B , Wlodawer A, Minor W et al Correcting the record of structural publications requires joint effort of the community and journal editors. FEBS J 2016;283:4452–7. 27229767 10.1111/febs.13765 PMC5124416

[vbaf156-B70] Schmidt D , Boehm M, McClendon CL et al Cosolvent-enhanced sampling and unbiased identification of cryptic pockets suitable for structure-based drug design. J Chem Theory Comput 2019;15:3331–43. 30998331 10.1021/acs.jctc.8b01295

[vbaf156-B71] Schmidt K-H et al Metadynamics: a method to simulate rare events and reconstruct the free energy in biophysics, chemistry and material science. Rep Progr Phys 2008;71:126601.

[vbaf156-B72] Seco J , Luque FJ, Barril X et al Binding site detection and druggability index from first principles. J Med Chem 2009;52:2363–71. 19296650 10.1021/jm801385d

[vbaf156-B73] Smith RD , Carlson HA. Identification of cryptic binding sites using MixMD with standard and accelerated molecular dynamics. J Chem Inf Model 2021;61:1287–99. 33599485 10.1021/acs.jcim.0c01002 PMC8091066

[vbaf156-B74] Sugita Y , Okamoto Y. Replica-exchange molecular dynamics method for protein folding. Chem Phys Lett 1999;314:141–51.

[vbaf156-B75] Sun Z , Wakefield AE, Kolossvary I et al Structure-based analysis of cryptic-site opening. Structure 2020;28:223–35.e2. 31810712 10.1016/j.str.2019.11.007 PMC7004864

[vbaf156-B76] Torrie GM , Valleau JP. Nonphysical sampling distributions in Monte Carlo free-energy estimation: umbrella sampling. J Comput Phys 1977;23:187–99.

[vbaf156-B77] Tze-Yang Ng J , Tan YS. Accelerated ligand-mapping molecular dynamics simulations for the detection of recalcitrant cryptic pockets and occluded binding sites. J Chem Theory Comput 2022;18:1969–81. 35175753 10.1021/acs.jctc.1c01177

[vbaf156-B78] Umezawa K , Kii I. Druggable transient pockets in protein kinases. Molecules 2021;26:651. 33513739 10.3390/molecules26030651 PMC7865889

[vbaf156-B79] Vajda S , Beglov D, Wakefield AE et al Cryptic binding sites on proteins: definition, detection, and druggability. Curr Opin Chem Biol 2018;44:1–8. 29800865 10.1016/j.cbpa.2018.05.003 PMC6088748

[vbaf156-B151] Verma N , Qu X, Trozzi F et al SSnet: a deep learning approach for protein-ligand interaction prediction. Int J Mol Sci 2021;22:1392. 33573266 10.3390/ijms22031392 PMC7869013

[vbaf156-B80] Wang Z , Harkins PC, Ulevitch RJ et al The structure of mitogen-activated protein kinase p38 at 2.1-Å resolution. Proc Natl Acad Sci U S A 1997;94:2327–32. 9122194 10.1073/pnas.94.6.2327 PMC20087

[vbaf156-B81] Whalen KL , Tussey KB, Blanke SR et al Nature of allosteric inhibition in glutamate racemase: discovery and characterization of a cryptic inhibitory pocket using atomistic MD simulations and p Ka calculations. J Phys Chem B 2011;115:3416–24. 21395329 10.1021/jp201037t PMC3072873

[vbaf156-B82] Wlodawer A , Dauter Z, Shabalin IG et al Ligand‐centered assessment of SARS‐CoV‐2 drug target models in the protein data bank. FEBS J 2020;287:3703–18. 32418327 10.1111/febs.15366 PMC7276724

[vbaf156-B83] Yanagisawa K , Moriwaki Y, Terada T et al EXPRORER: rational cosolvent set construction method for cosolvent molecular dynamics using large-scale computation. J Chem Inf Model 2021;61:2744–53. 34061535 10.1021/acs.jcim.1c00134

[vbaf156-B84] Yang W-C , Gong D-H, Gao Y-Y et al Grasping cryptic binding sites to neutralize drug resistance in the field of anticancer. Drug Discov Today 2023;28:103705. 37453458 10.1016/j.drudis.2023.103705

[vbaf156-B85] Zheng W. Predicting cryptic ligand binding sites based on normal modes guided conformational sampling. Proteins 2021;89:416–26. 33244830 10.1002/prot.26027

