# Supplementary material for: Recent computational advances in the identification of cryptic binding sites for drug discovery
Source: Bioinform Adv. 2025 Jul 1;5(1):vbaf156. doi: 10.1093/bioadv/vbaf156 (PMC12342141; doi:10.1093/bioadv/vbaf156)
Supplement: vbaf156_Supplementary_Data [file vbaf156_supplementary_data.docx]

*Supplementary Information*

*
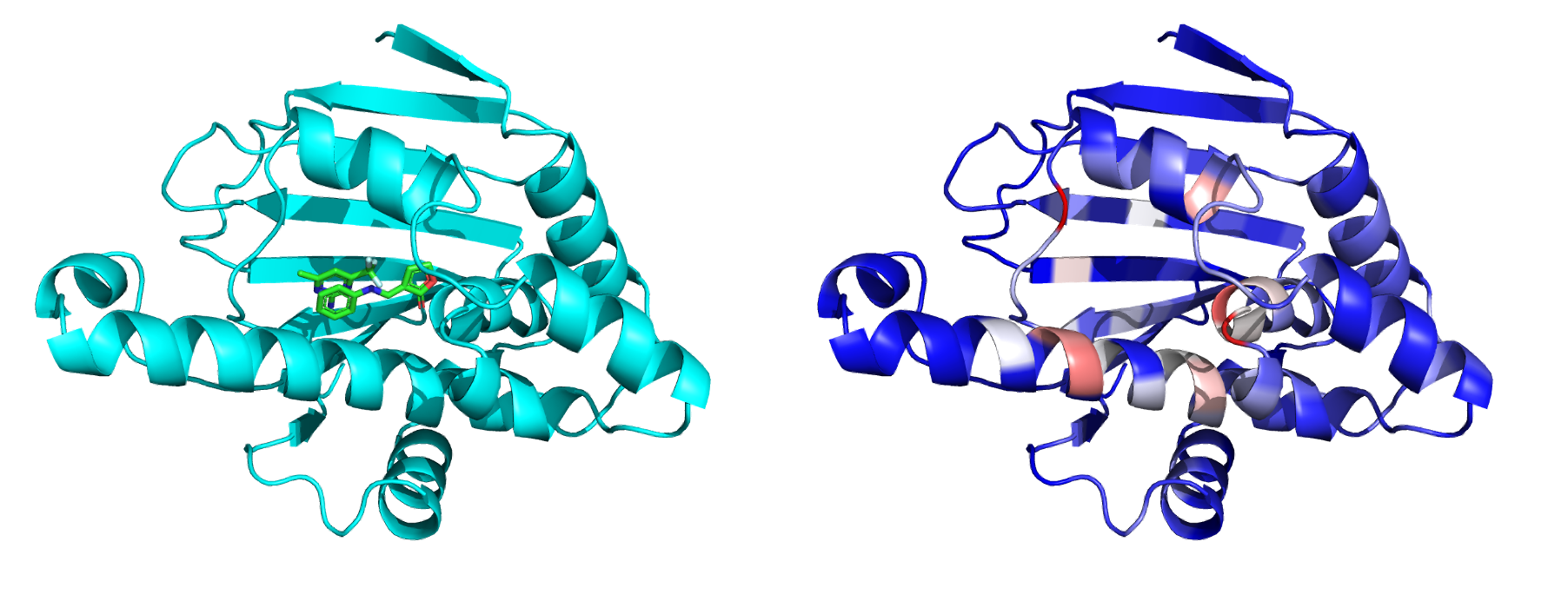
* **Supplementary Figure 1***: Ribbon diagram of Hsp90 (PDB entry: 2qfo) with the CryptoSite evaluation on the right. Shades of red indicate a higher probability of amino acid residue contribution to a cryptic binding site. Shades of blue indicate a lower probability of an amino acid residue participating in a cryptic binding site. The bound ligand is depicted in green stick representation.*

*
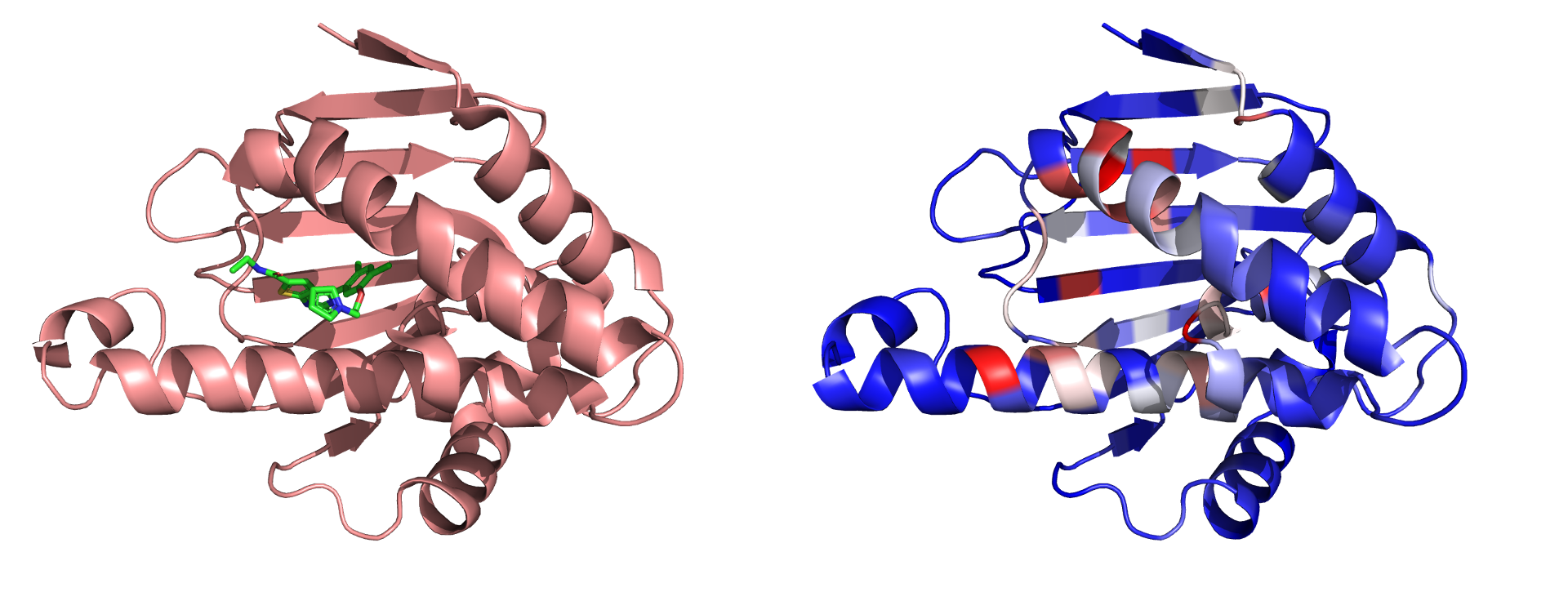
*

**Supplementary Figure 2***: Ribbon diagram of Hsp90 (PDB entry: 2wi7) with the CryptoSite evaluation on the right. Shades of red indicate a higher probability of amino acid residue contribution to a cryptic binding site. Shades of blue indicate a lower probability of an amino acid residue participating in a cryptic binding site. The bound ligand is depicted in green stick representation.*


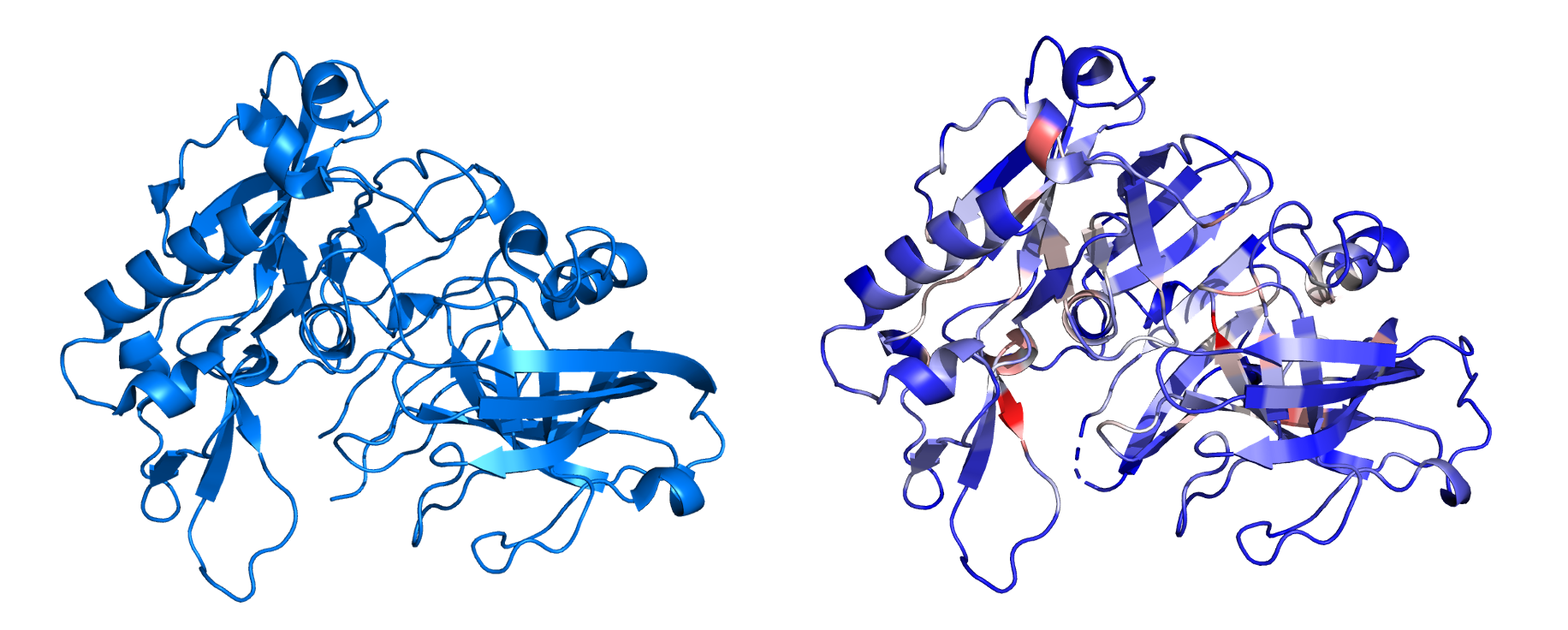


**Supplementary Figure 3:** *Ribbon diagram of BACE-1 (PDB entry: 1w50) with the CryptoSite evaluation on the right. Shades of red indicate a higher probability of amino acid residue contribution to a cryptic binding site. Shades of blue indicate a lower probability of an amino acid residue participating in a cryptic binding site.*

*
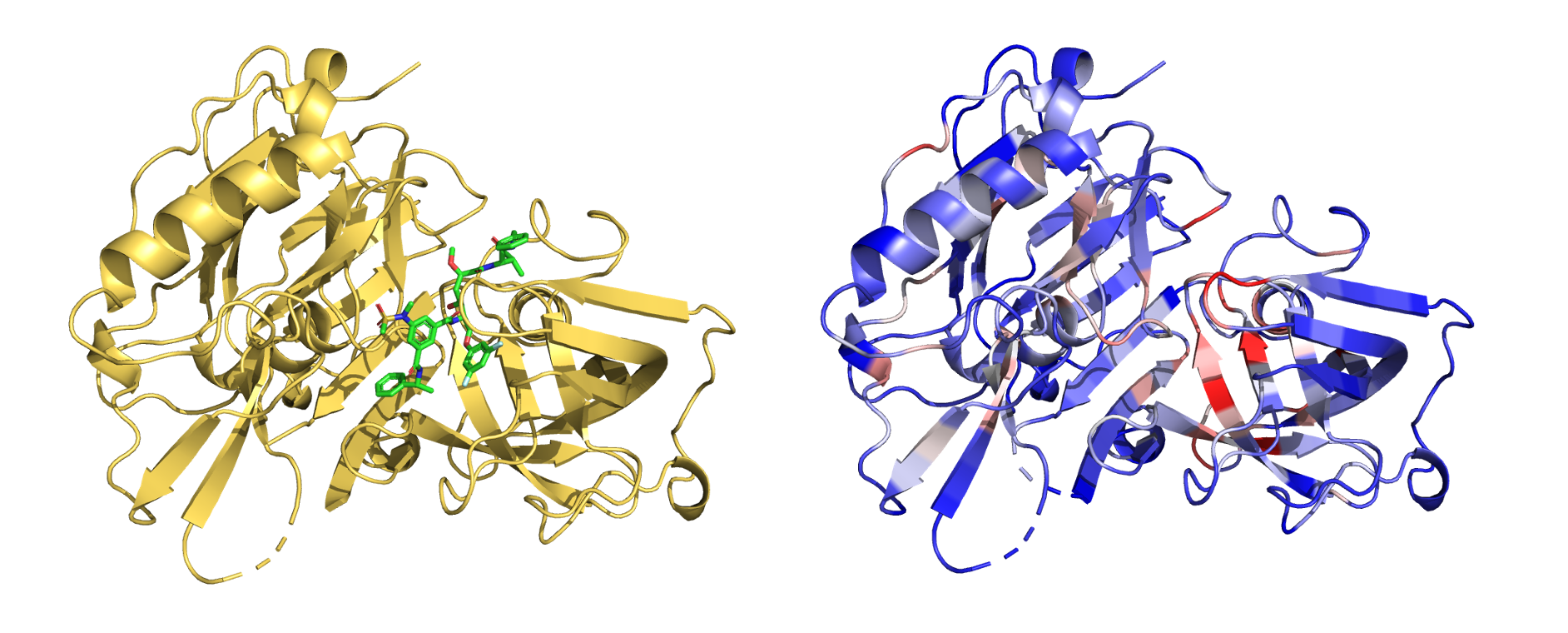
*

**Supplementary Figure 4:** *Ribbon diagram of BACE-1 (PDB entry: 3ixj) with the CryptoSite evaluation on the right. Shades of red indicate a higher probability of amino acid residue contribution to a cryptic binding site. Shades of blue indicate a lower probability of an amino acid residue participating in a cryptic binding site. The bound ligand is depicted in green stick representation.*

*
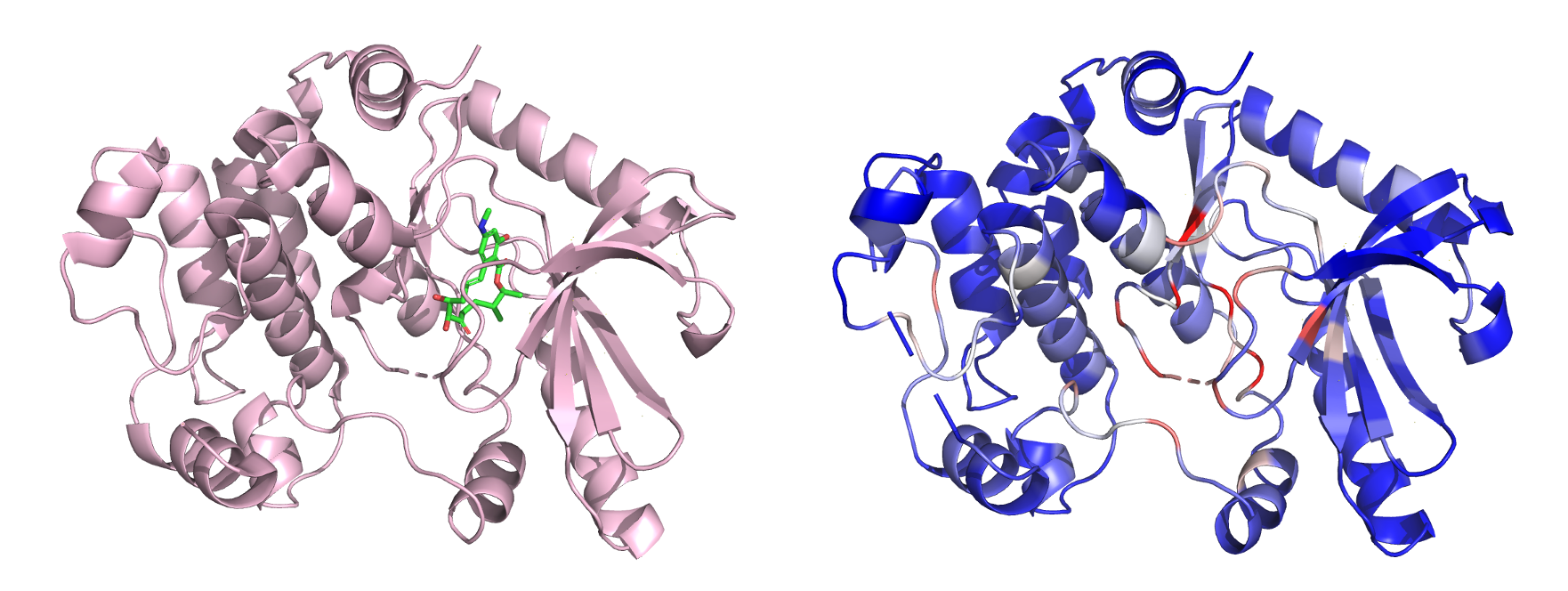
*

**Supplementary Figure 5:** *Ribbon diagram of Mek-1 (PDB entry: 5hze) with the CryptoSite evaluation on the right. Shades of red indicate a higher probability of amino acid residue contribution to a cryptic binding site. Shades of blue indicate a lower probability of an amino acid residue participating in a cryptic binding site. The bound ligand is depicted in green stick representation.*

*
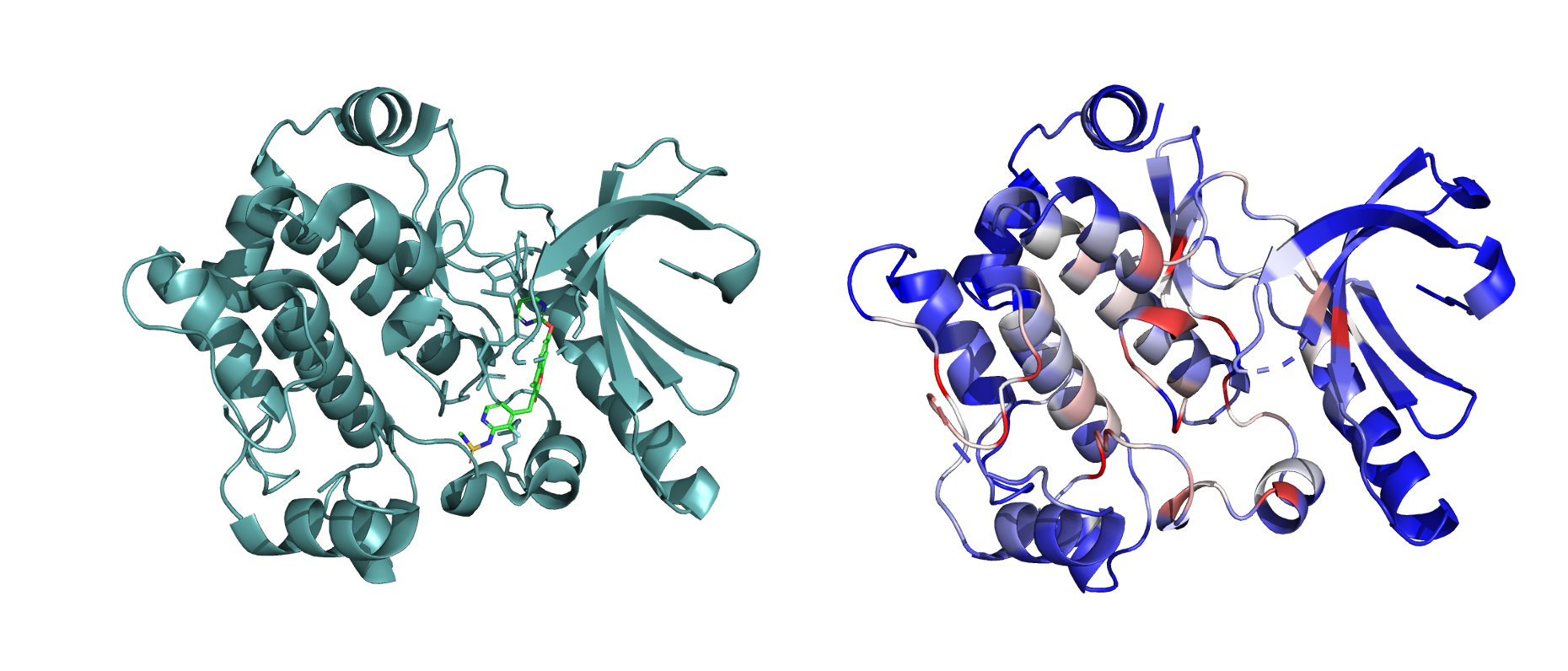
*

**Supplementary Figure 6:** *Ribbon diagram of Mek-1 (PDB entry: 3wig) with the CryptoSite evaluation on the right. Shades of red indicate a higher probability of amino acid residue contribution to a cryptic binding site. Shades of blue indicate a lower probability of an amino acid residue participating in a cryptic binding site. The bound ligand is depicted in green stick representation.*

**
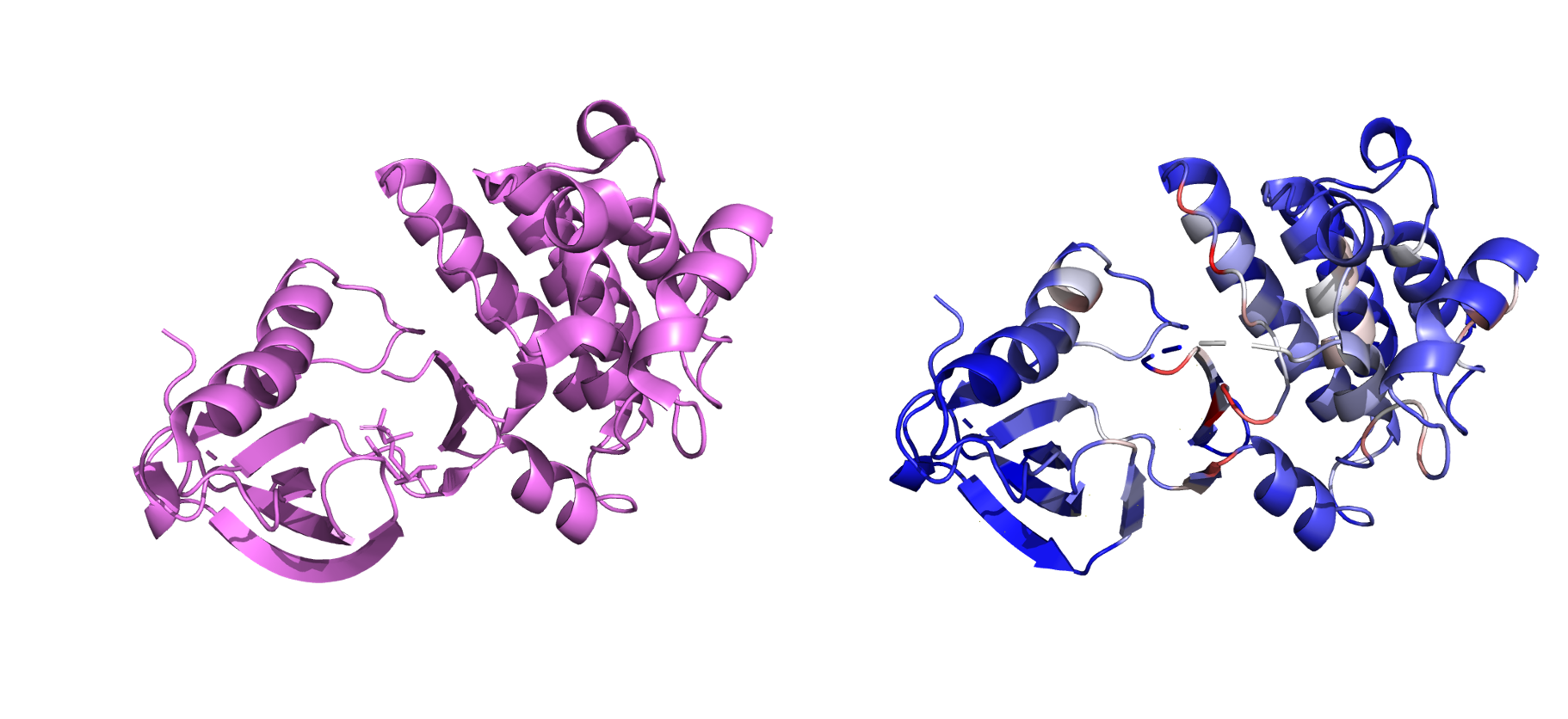
 Supplementary Figure 7:** *Ribbon diagram of FAK (PDB entry: 1mpe) with the CryptoSite evaluation on the right. Shades of red indicate a higher probability of amino acid residue contribution to a cryptic binding site. Shades of blue indicate a lower probability of an amino acid residue participating in a cryptic binding site.*


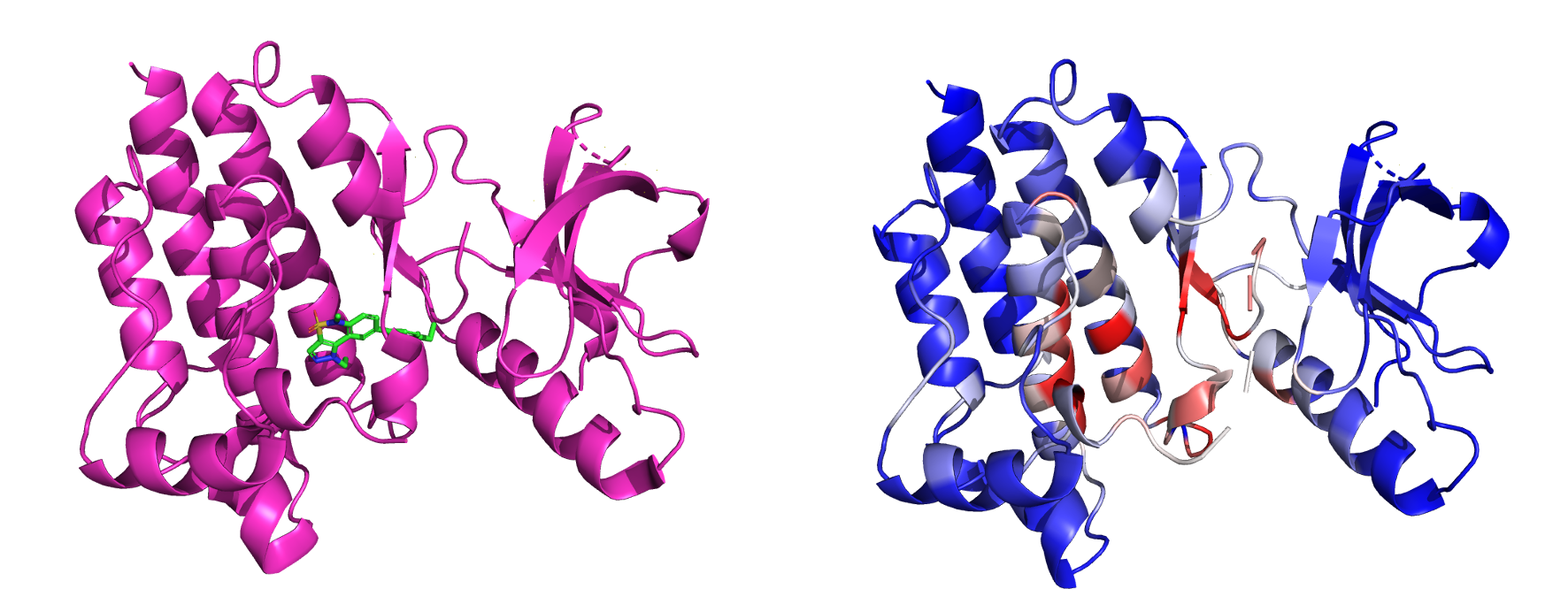


**Supplementary Figure 8:** *Ribbon diagram of FAK (PDB entry: 4ebw) with the CryptoSite evaluation on the right. Shades of red indicate a higher probability of amino acid residue contribution to a cryptic binding site. Shades of blue indicate a lower probability of an amino acid residue participating in a cryptic binding site. The bound ligand is depicted in green stick representation.*


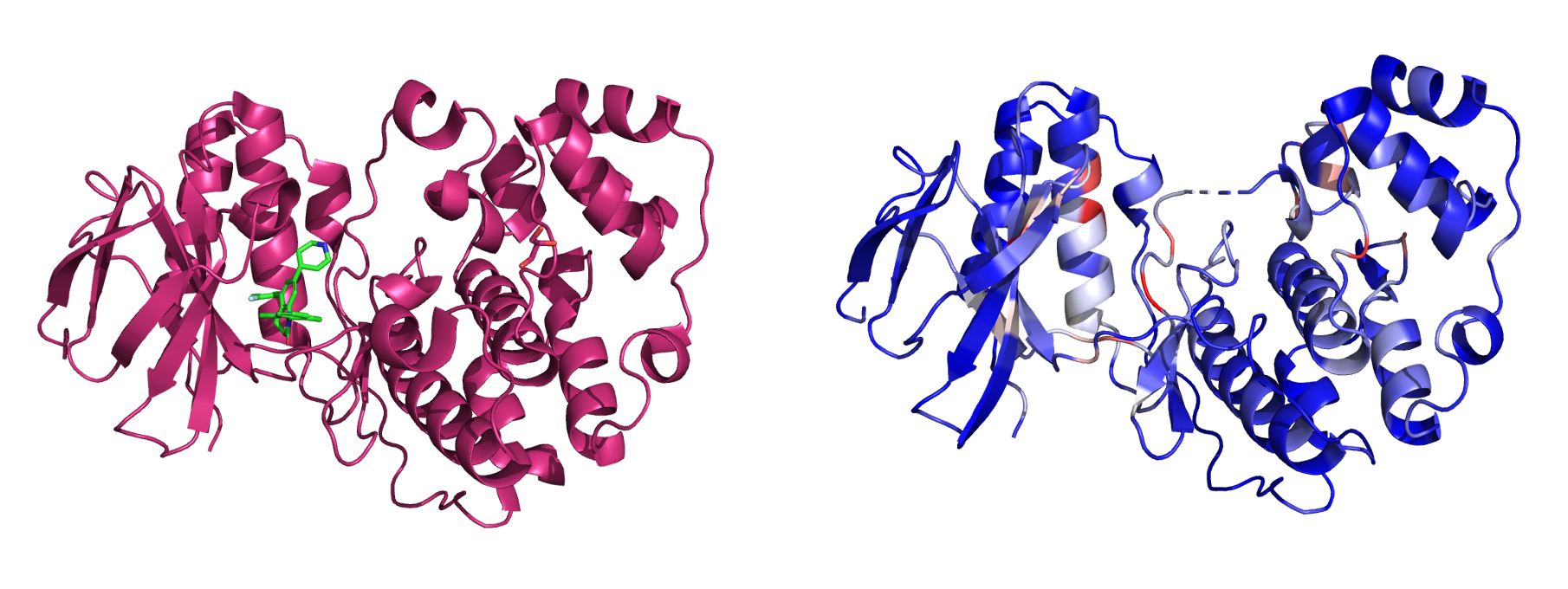
 **Supplementary Figure 9:** *Ribbon diagram of p38 (PDB entry: 1ove) coloured according to CryptoSite scoring. Shades of red indicate a higher probability of amino acid residue contribution to a cryptic binding site. Shades of blue indicate a lower probability of an amino acid residue participating in a cryptic binding site. The bound ligand is depicted in green stick representation.*


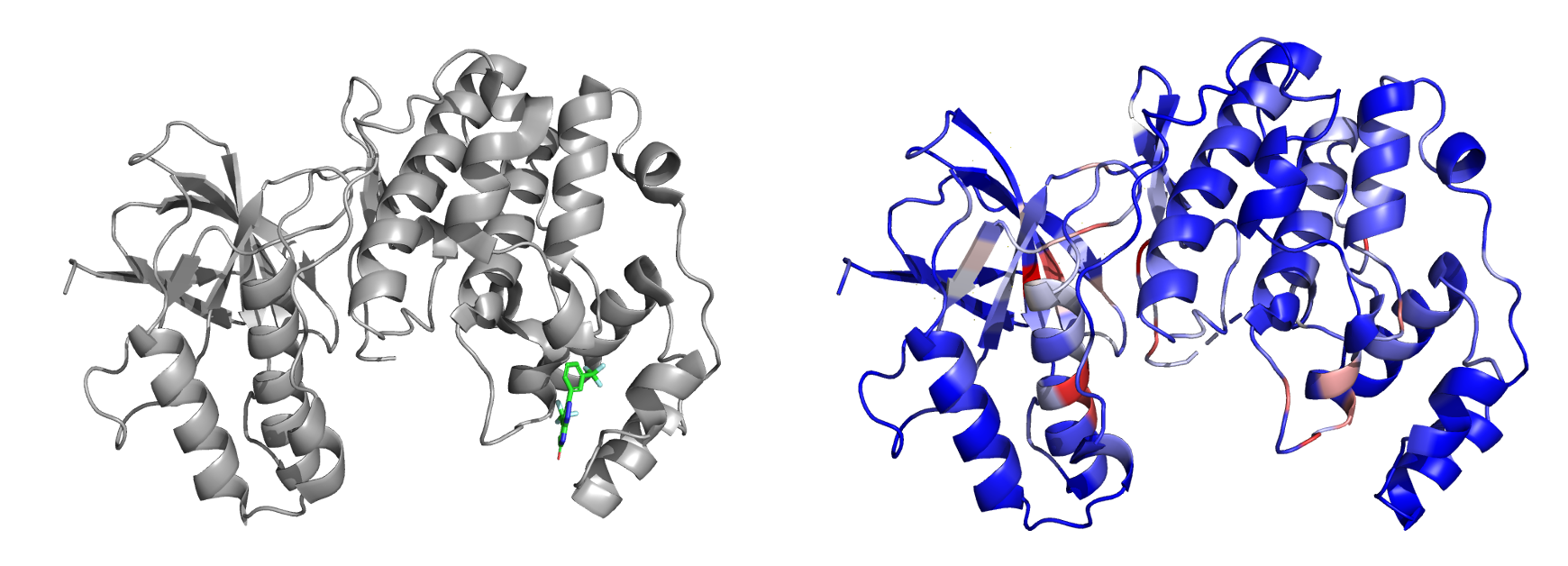


**Supplementary Figure 10:** *Ribbon diagram of p38 (PDB entry: 3new) coloured according to CryptoSite scoring. Shades of red indicate a higher probability of amino acid residue contribution to a cryptic binding site. Shades of blue indicate a lower probability of an amino acid residue participating in a cryptic binding site. The bound ligand is depicted in green stick representation.*

*
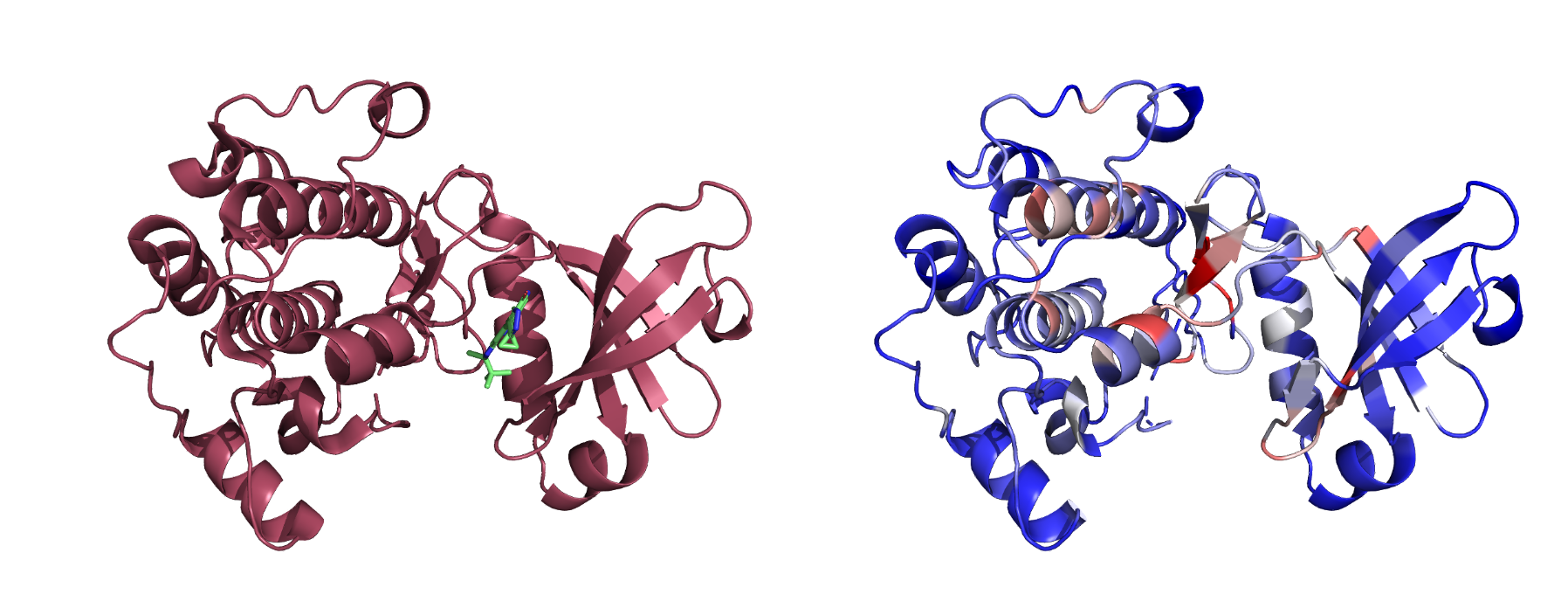
*

**Supplementary Figure 11:** *Ribbon diagram of PDK1* *(PDB entry: 3nay) coloured according to CryptoSite scoring. Shades of red indicate a higher probability of amino acid residue contribution to a cryptic binding site. Shades of blue indicate a lower probability of an amino acid residue participating in a cryptic binding site. The bound ligand is depicted in green stick representation.*


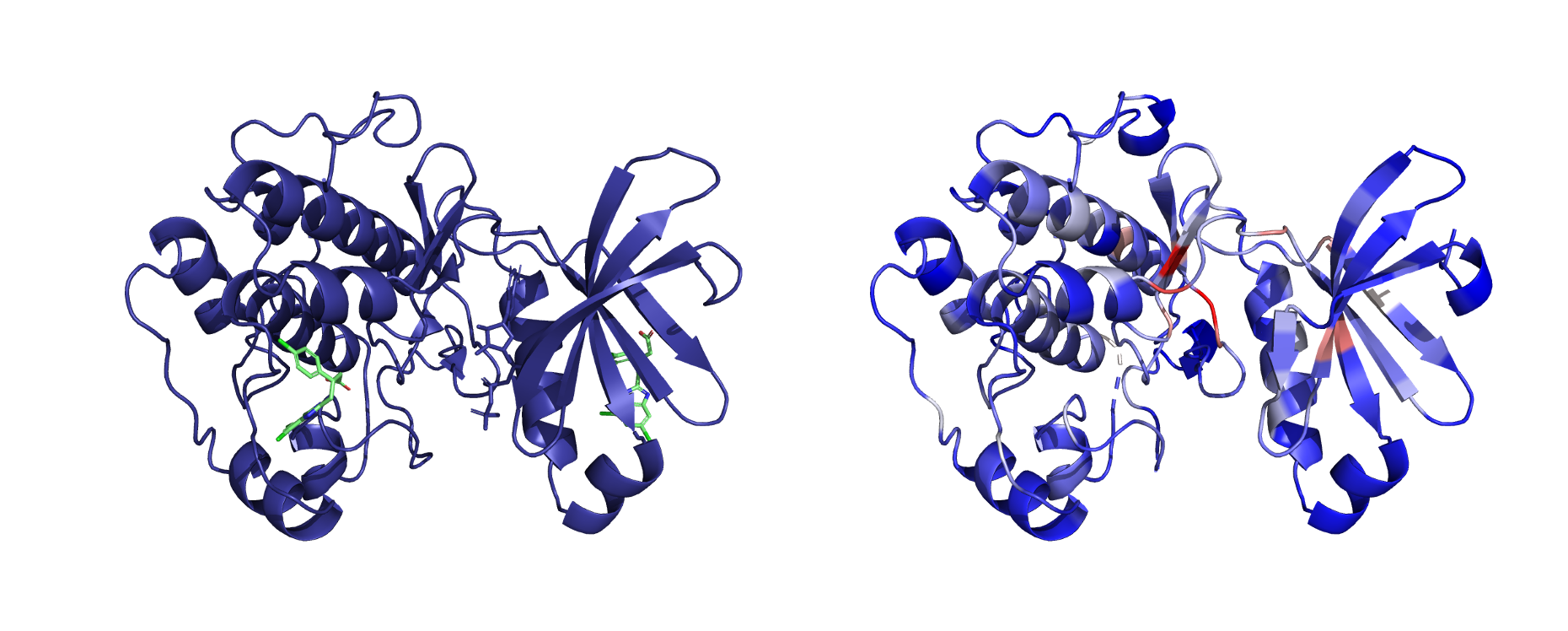


**Supplementary Figure 12:** *Ribbon diagram of PDK1* *(PDB entry: 4a07) coloured according to CryptoSite scoring. Shades of red indicate a higher probability of amino acid residue contribution to a cryptic binding site. Shades of blue indicate a lower probability of an amino acid residue participating in a cryptic binding site. The bound ligand is depicted in green stick representation.*


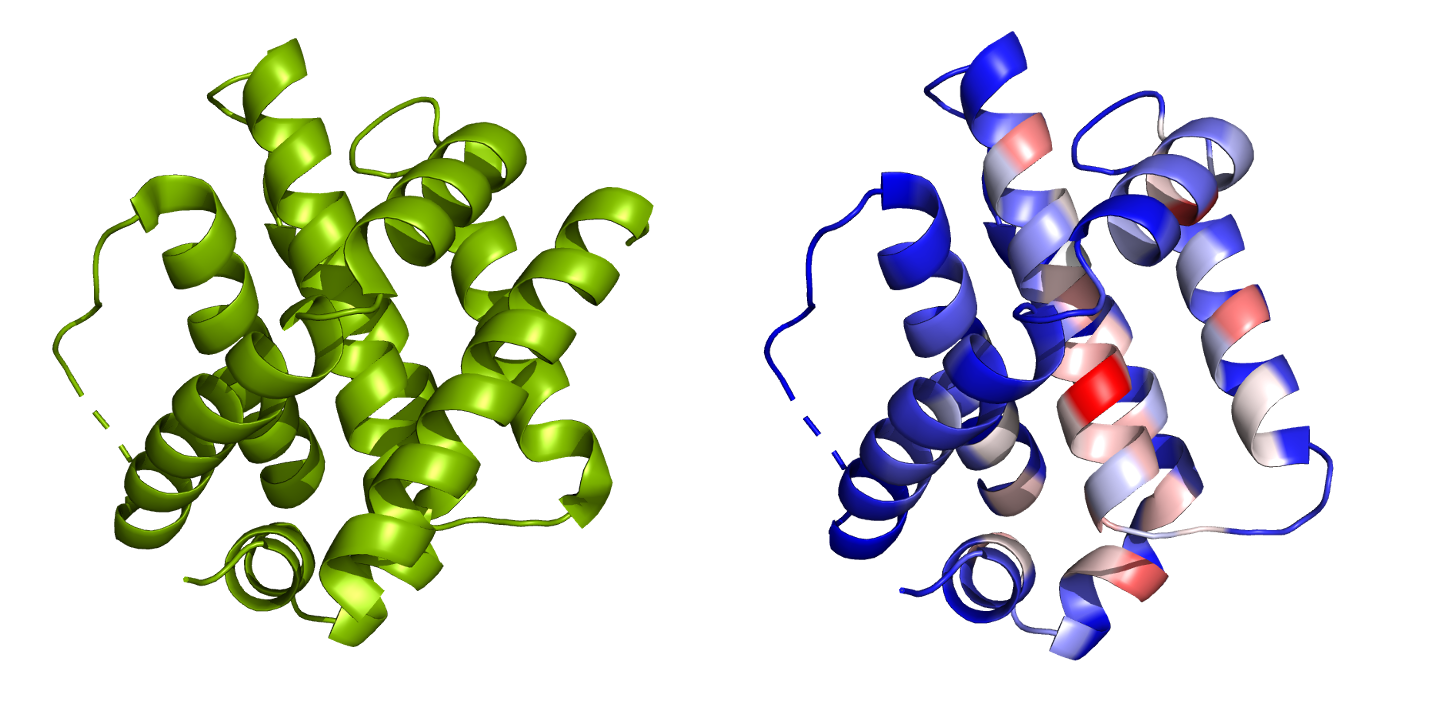


**Supplementary Figure 13:** *Ribbon diagram of McI-1* *(PDB entry: 2pqk) coloured according to CryptoSite scoring. Shades of red indicate a higher probability of amino acid residue contribution to a cryptic binding site. Shades of blue indicate a lower probability of an amino acid residue participating in a cryptic binding site.*


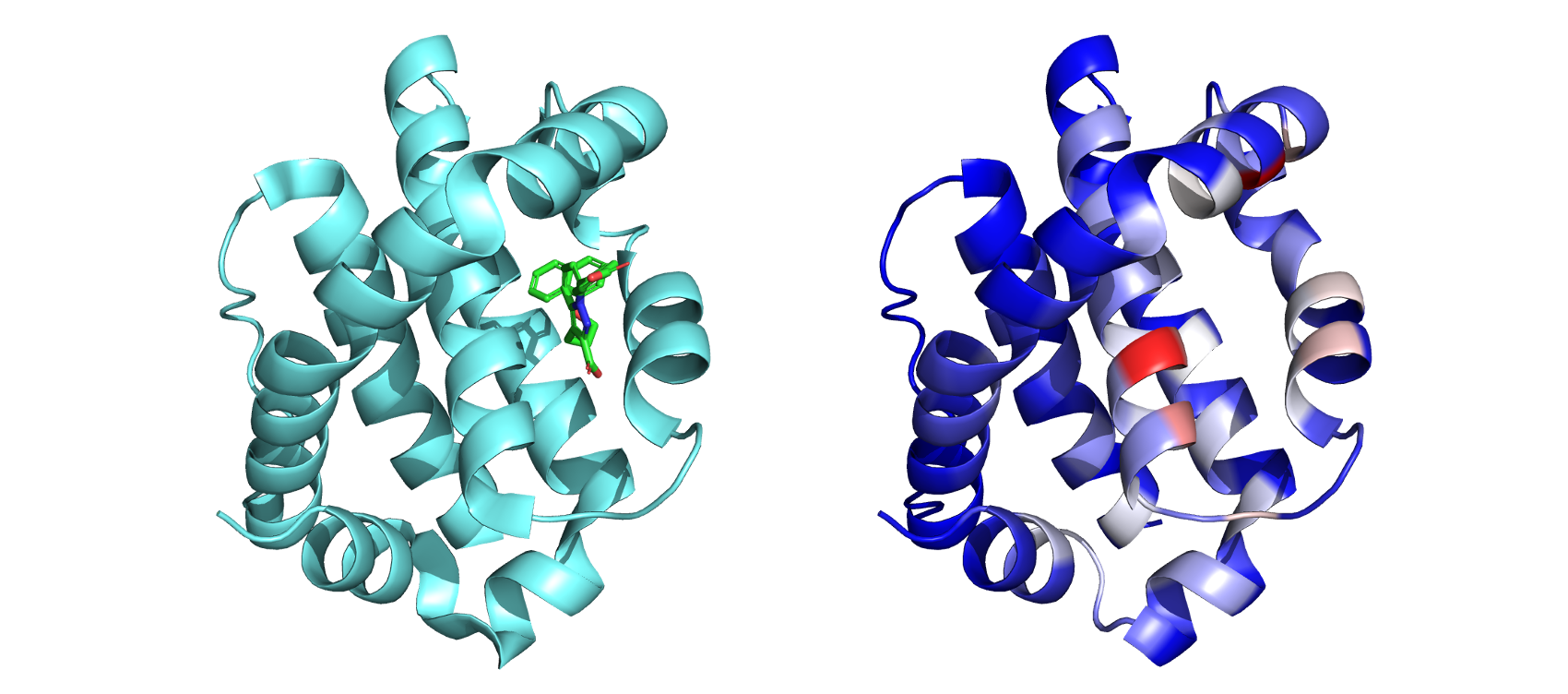
 **Supplementary Figure 14:** *Ribbon diagram of McI-1* *(PDB entry: 3wix) coloured according to CryptoSite scoring. Shades of red indicate a higher probability of amino acid residue contribution to a cryptic binding site. Shades of blue indicate a lower probability of an amino acid residue participating in a cryptic binding site. The bound ligand is depicted in green stick representation.*
